# Rejuvenation of BMSCs senescence by pharmacological enhancement of TFEB-mediated autophagy alleviates aged-related bone loss and extends lifespan in middle aged mice

**DOI:** 10.1038/s41413-024-00351-7

**Published:** 2024-08-21

**Authors:** Ziwei Luo, Wanyi Wei, Dawei Qiu, Zixia Su, Liangpu Liu, Honghai Zhou, Hao Cui, Li Yang

**Affiliations:** 1 College of Orthopedics, Guangxi University of Chinese Medicine, Nanning, 530200 Guangxi China; 2 Faculty of Chinese Medicine Science, Guangxi University of Chinese Medicine, Nanning, 530200 Guangxi China; 3 Department of Physical Education, Guangxi University of Chinese Medicine, Nanning, 530200 Guangxi China; 4 Guangxi Key Laboratory of Efficacy Study on Chinese Materia Medica, Guangxi University of Chinese Medicine, Nanning, 530200 Guangxi China; 5 College of Pharmacy, Guangxi University of Chinese Medicine, Nanning, 530200 Guangxi China; 6 Key Laboratory of Biorheological Science and Technology, Ministry of Education, Bioengineering College, Chongqing University, Chongqing, 400030 China

**Keywords:** Bone, Homeostasis, Bone quality and biomechanics

## Abstract

Bone marrow stromal/stem cells (BMSCs) are generally considered as common progenitors for both osteoblasts and adipocytes in the bone marrow, but show preferential differentiation into adipocytes rather than osteoblasts under aging, thus leading to senile osteoporosis. Accumulated evidences indicate that rejuvenation of BMSCs by autophagic enhancement delays bone aging. Here we synthetized and demonstrated a novel autophagy activator, CXM102 that could induce autophagy in aged BMSCs, resulting in rejuvenation and preferential differentiation into osteoblasts of BMSCs. Furthermore, CXM102 significantly stimulated bone anabolism, reduced marrow adipocytes, and delayed bone loss in middle-age male mice. Mechanistically, CXM102 promoted transcription factor EB (TFEB) nuclear translocation and favored osteoblasts formation both in vitro and in vivo. Moreover, CXM102 decreased serum levels of inflammation and reduced organ fibrosis, leading to a prolonger lifespan in male mice. Our results indicated that CXM102 could be used as an autophagy inducer to rejuvenate BMSCs and shed new lights on strategies for senile osteoporosis and healthyspan improvement.

## Introduction

Bone marrow stromal/stem cells (BMSCs) are generally considered as the common progenitors for both osteoblasts and adipocytes in bone marrow, and have been greatly shown for clinical application potential.^1^ However, their number and function decline with aging, especially the preferential differentiation of aged BMSCs into adipocytes rather than osteoblasts is reasonably accepted as a leading cause of senile osteoporosis (SOP), which is characterized by increased bone marrow fat accumulation and decreased bone loss.^2–4^ Thus, the balance between osteogenic and adipogenic lineage commitment of BMSCs is essential for bone homeostasis. Despite the mechanisms under which the lineage shift occurs in aged BMSCs are not fully clear, accumulated studies have showed that diversity strategies for BMSCs rejuvenation are of benefits for bone quality and even healthspan improvement.^5–13^ For example, modification of transcription factors,^6,7^ epigenetics^10–14^ and autophagy^5,15,16^ that enhanced osteogenesis and decreased adipogenesis of BMSCs alleviated SOP in mice. The latest evidence uncovered that premature aging of skeletal stem/progenitor cells caused bone loss.^17^ Therefore, it is assumed that stimulation of bone formation by BMSCs rejuvenation in vivo is an effective and attractive strategy for age-associated bone loss.^18^ Although the only currently approved anabolic agent-teriparatide-a recombinant parathyroid hormone (rPTH), has been shown to stimulate BMSCs osteogenesis and suppress adipogenesis in a variety of manners,^19–24^ several adverse factors, such as progressive blunting of bone formation after 18–24 months treatment, decreased osteoblast precursors and increased osteosarcoma risks, limit its widespread use.^18,23^

Autophagy is a complex dynamic process involved in the degradation and correction of intracellular damaged proteins and organelles, playing a vital role in bone and other tissue homeostasis.^16,25,26^ Increasing evidence showed that compromised autophagy is considered as one hallmark of aging and associated with aged-related diseases, including osteoporosis.^16,25–29^ A previous study also demonstrated that suppression of autophagy in osteocytes resulted in skeletal aging.^30^ In the bone marrow, autophagy is required for BMSCs homeostasis and plays a fundamental role in the onset and progression of pathological osteoporosis.^26^ Decreased autophagy has been shown in aged BMSCs, while enhanced autophagy by rapamycin could direct osteogenic lineage of aged BMSCs and rescued bone loss.^31,32^ Therefore, targeting autophagy that can rejuvenate stem cells is one of the most promising strategies for geroprotection.^33^ Although rapamycin has been served as a golden standard for autophagy induction and shown exciting results in clinical trials, the adverse effects limit its widely used in the treatment of SOP and other age-related diseases.^34–36^ Hence, more safer autophagy agonists are needed to be explored.

Transcription factor EB (TFEB) is a key transcriptional regulator of autophagy and lysosomal biogenesis. Emerging discoveries demonstrated that TFEB overexpression promoted longevity and reduced the burden of diseases, holding great promise as a therapeutic strategy for multiple age-associated diseases.^37,38^ Regulation of TFEB has been shown to control the activities of osteoblasts and osteoclasts,^39,40^ the two main cells playing in the coupling of bone remodeling for homeostasis, implying its potential use in osteoporosis prevention. However, little is known about the relationship between TFEB activities and osteoporosis. As precursors of bone lineage cells, BMSCs directly contribute to bone remodeling by differentiating into osteoblasts, but how and to what extend TFEB regulates fate decision of aged BMSCs in bone marrow is still unclear. Beyond its photodynamic therapy, verteporfin also shows autophagy inhibition,^41^ but little is known about the detailed effects on different steps involved in autophagy and fate decision of BMSCs.

In this study, we synthesized a novel small molecule compound (named “CXM102”) that could promote autophagy activities in aged BMSCs via enhancement of TFEB nuclear translocation, leading to senescence rejuvenation and bone anabolic effects in middle age mice. Additionally, low dose and long-term administration of CXM102 showed better benefits for healthspan than rapamycin in mice, including extended lifespan, reduced serum levels of inflammation, less lipid droplets and fibrosis in organs. Our results demonstrated that CXM102 could significantly counteract aberrant lineage allocations of aged BMSCs, alleviate osteoporotic bone loss, increase healthspan and longevity of middle age mice.

## Results

### CXM102 induces autophagic influx in hBMSCs

**Fig. 1:**
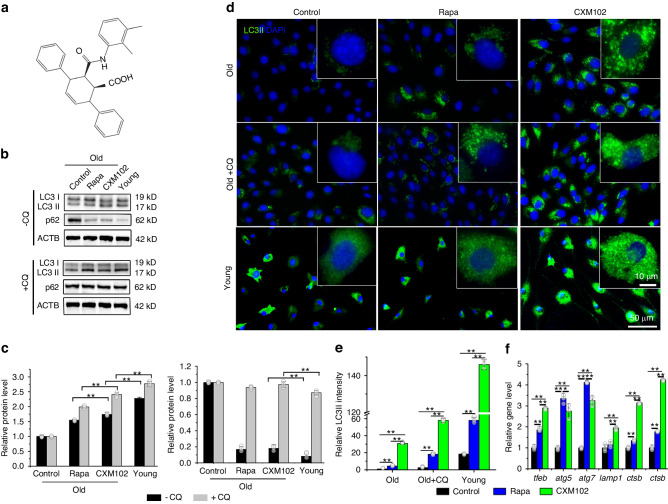
CXM102 increases autophagic influx in hBMSCs. **a** Structure of CXM102. Western blotting (**b**) and quantitative (**c**) analysis of autophagy influx in young and old hBMSCs stimulated by DMSO (Control), CXM102 or rapamycin with CQ or not. Represent images (**d**) and relative intensity (**e**) of LC3II puncta detection in young and old hBMSCs treated with DMSO (Control), CXM102, rapamycin or CQ. **f** Autophagy and lysosome related gene analysis in old BMSCs treated with DMSO (Control), CXM102 or rapamycin. Results are presented as means ± SD, *n* ≥ 3, *P < 0.05, ** *P* < 0.01

As AMPK activators, arctigenin and its derivatives show remarkable therapeutic potential against a wide range of human diseases.^42,43^ But from an unsuccessful experience of synthesis of arctigenin derivatives, we tried to redesign and synthesize a novel compound (Figs. S1a and 1a, hydrogen spectrum in Fig. S1b). Then the bioactivities were screened by cell viability. CXM102 had no cytotoxic effects on several normal cells, including BMSCs, liver cells (L02), synoviocytes and skin fibroblasts (FEK4) (Fig. S1c), but exhibited significant inhibition of cell growth in many cancer cell lines (Hep3B, HCCLM3, HCT116, DU145, A549 and THP-1) and rheumatoid arthritis fibroblast-like synoviocytes (RA-FLS) in a dose-dependent manner (Fig. S1d), suggesting its bidirectional effects on cell growth in human normal cells and cancer cells. Given the roles of arctigenin in activation of AMPK pathway and mesenchymal stem cell fate regulation,^43,44^ we tried to study the effects of CXM102 on autophagy activities in young and old hBMSCs by comparing with rapamycin. Interestingly, CXM102 significantly increased autophagic activities in both young and old hMSCs, as revealed by elevated levels of LC3II and decreased p62 (Fig. 1b, c). Considering that activation of autophagy can delay cellular senescence in BMSCs,^32^ we analyzed the effect of CXM102 on autophagic flux in old hBMSCs treated with chloroquine (CQ). Compared with rapamycin, CXM102 increased higher autophagic influx in old hBMSCs (Fig. 1b–e). Additionally, autophagy related genes (*Atg5, Atg7*) and lysosome related genes (*Lamp1, Ctsb, Ctsd*) were also upregulated by CXM102, accompanied with the elevated expression of upstream regulatory gene *Tfeb* (Fig. 1f). These results demonstrated that CXM102 could increase autophagy in young and old hBMSCs.

### CXM102 ameliorates cellular senescence of hBMSCs in vitro

**Fig. 2:**
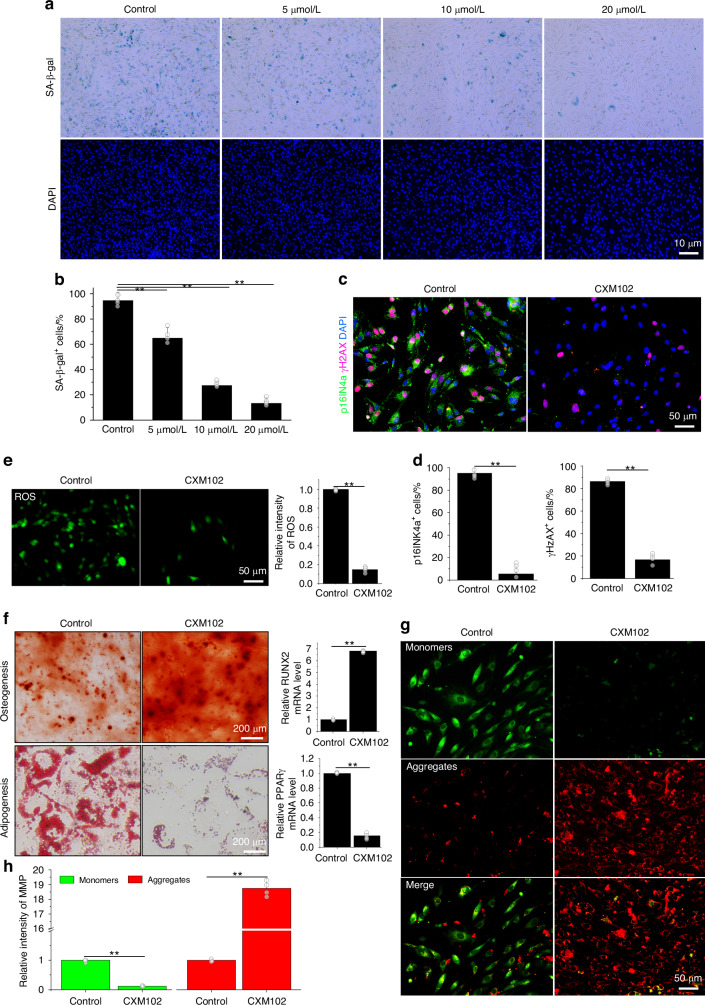
CXM102 ameliorates the senescence-associated phenotypes of hBMSCs. Representative images (**a**) and quantitative analysis (**b**) of SA-β-gal and DAPI staining. Representative images (**c**) and quantitative analysis (**d**) of double immunofluorescent staining of p16INK4a and γH2AX. **e** Representative images and quantitative analysis of intracellular ROS level. **f** Representative images and quantitative analysis of relative gene levels of *Runx2* and *Pparγ* for osteogenic and adipogenic differentiation with or without CXM102 treatment. Representative images (**g**) of JC-1 staining and quantitative analysis and intensity analysis of mitochondrial membrane potential (MMP) (**h**) in **g**. Results are presented as means ± SD, *n* ≥ 3. **P < 0.01

It is widely accepted that autophagy declines with age in BMSCs and activation of autophagy could alleviate cellular senescence.^15,32^ Then we explored the inducible autophagic effects of CXM102 on senescent hBMSCs. Aged BMSCs are characterized by elevated senescence-associated β-galactosidase (SA-β-gal) activities, levels of cyclin-dependent kinase 2a (CDK2a/p16INK4a), phosphorylation of Ser-139 of histone H2A.X (γH2A.X), reactive oxygen species (ROS) and adipogenic bias, but accompanied with declined autophagy, mitochondrial membrane potential (MMP) and osteogenic bias (Fig. S2), which were consistent with others’ works.^3,45^ However, CXM102 increased autophagosomes, but reduced SA-β-gal activities, levels of p16INK4a, γH2A.X, ROS and adipogenesis, while restored the MMP and osteogenesis in old hBMSCs (Fig. S2). To confirm the effects of CXM102 on senescence alleviation, we used hydrogen peroxide (H_2_O_2_) to induce cellular senescence in a hBMSCs cell line. As revealed by SA-β-gal staining (Fig. 2a), 2-h exposure of 200 μmol/L H_2_O_2_ was successfully to induce cellular senescence, but pretreatment with CXM102 led to significantly decreased SA-β-gal activities in a dose-dependent manner (Fig. 2a, b). Besides, CXM102 also downregulated levels of p16INK4a, γH2A.X, ROS (Fig. 2c–e). In addition, CXM102 promoted osteogenic and reduced adipogenic differentiation of hBMSCs (Fig. 2f). To detect the effect of CXM102 on mitochondrial integrity, we conducted MMP detection based on JC-1 staining^46,47^ and found that CXM102 could remarkably restored the lost MMP (Fig. 2g, h). However, pretreatment with 3-methyladenine (3-MA) blocked the CXM102-induced autophagy assembly and failed to rejuvenate old hBMSCs, as charactered by elevated SA-β-gal activities, levels of p16INK4a and γH2A.X, ROS and decreased MMP and osteogenic differentiation (Fig. S2). Taken together, these data demonstrated that CXM102-induced autophagy could rejuvenate aged hBMSCs in vitro.

### CXM102 induces autolysosomes formation via TFEB nuclear translocation in aged hBMSCs

**Fig. 3:**
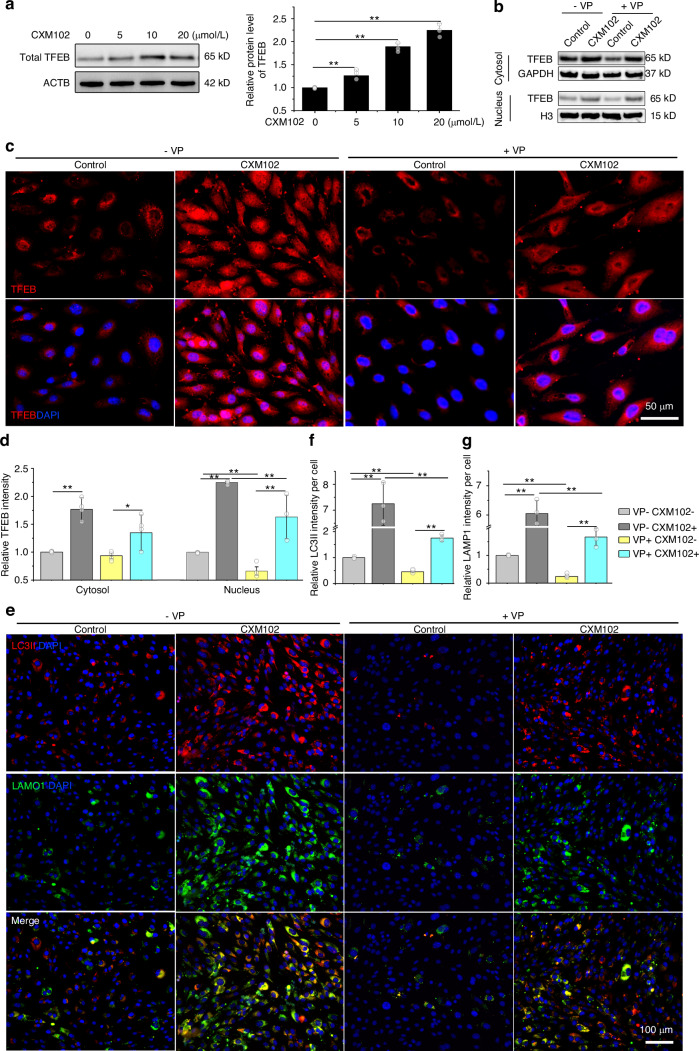
CXM102 induces autolysosomes formation via TFEB nuclear translocation in hBMSCs. **a** Western blotting analysis of total TFEB expression treated with or without CXM102. Representative cytosol and nucleus TFEB analysis (**b**), immunofluorescent staining for sublocation (**c**) and relative quantitative analysis (**d**) of TFEB treated with or without CXM102. **e** Representative fluorescent images of double staining of LC3II and LAMP1. **f**, **g** Quantitative analysis of average LC3II and LAMP1 per cell in **c** Results are presented as means ± SD, n = 3. *P < 0.05, **P < 0.01

Subsequently, we investigated the upstream regulators that control the autophagic activation by CXM102. As CXM102 could upregulate *Tfeb* and autophagy and lysosome related genes (Fig. 1f), we tried to investigate the protein level of TFEB, which has been considered as a master regulator of lysosomal biogenesis and autophagy.^38,48,49^ As expected, CXM102 upregulated total TFEB expression in aged hBMSCs (Fig. 3a). Given that the transcriptional activity of TFEB mainly depends on its cellular localization, we found that CXM102 remarkedly increased nuclear translocation of TFEB (Fig. 3b–d). To confirm the role of TFEB in CXM102-induced autophagy, we conducted siRNA and pharmacological inhibitors to explore the activities of TFEB. Transfection with siTFEB could downregulate *Tfeb* and its downstream genes expression within 24 h, but not in 72 h (Fig. S3a). This data was consistent with the results from immunoblotting and immunofluorescent experiments (Fig. S3b, c). These results suggested that siTFEB transfection failed to inhibit nuclear translocation of TFEB for long-term experiments. However, we screened an inhibitor, verteporfin (VP), for the special regulation of nuclear translocation of TFEB. VP didn’t change the global gene and protein levels of TFEB (Fig. S3a, b), but inhibit its nuclear translocation (Fig. 3b–d) and downstream genes for 72 h (Fig. S3a). Interestingly, CXM102 partially rescued the VP-induced inhibition of TFEB translocation into nucleus (Fig. 3c, d). In accordance with the TFEB activities regulated by CXM102 and VP, markers for autophagosomes (LC3II) and lysosomes (LAMP1) exhibited similar expression (Fig. 3e–g). Correspondingly, VP treatment led to deteriorated senescence of old hBMSCs, including higher SA-β-Gal activities, γH2AX and p16INK4a positive cells and lower MMP (Fig. S3d–f). These results demonstrated that CXM102 induced autophagy via TFEB nuclear translocation to suppress hBMSCs senescence.

### CXM102 regulates lineage choice of hBMSCs through nuclear translocation of TFEB

**Fig. 4:**
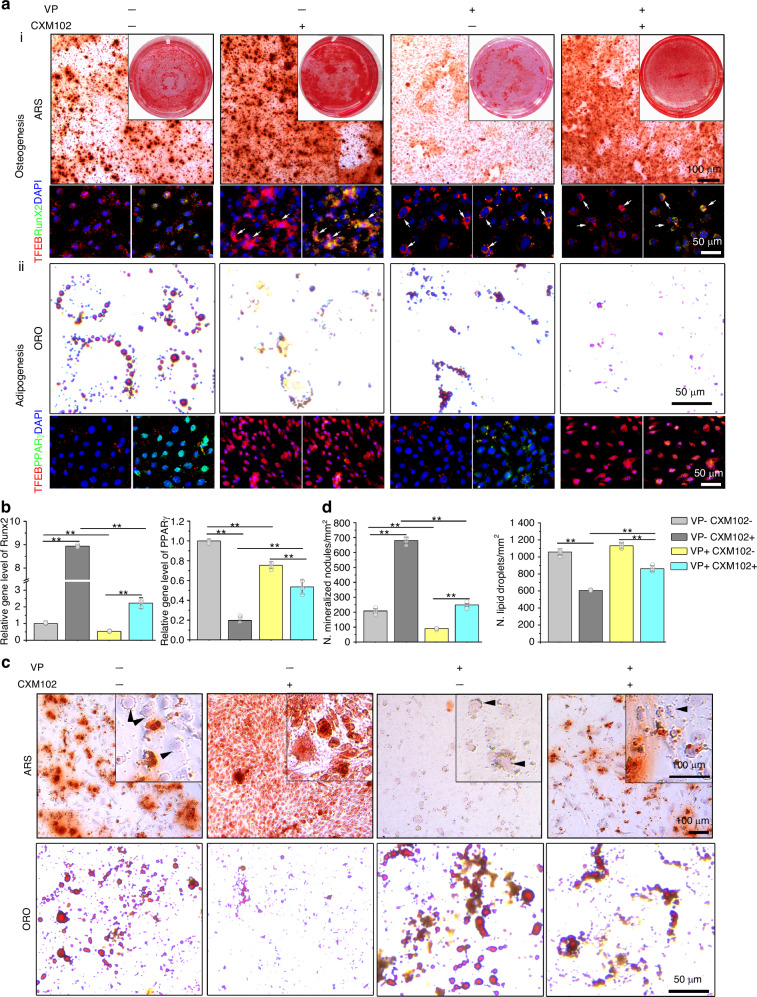
CXM102 remodels the bone-fat differentiation of young and aged hBMSCs in vitro. **a** Representative images of young hBMSCs responded to (i) osteogenic (ARS staining) or (ii) adipogenic (ORO staining) induction treated with or without verteporfin (VP) and CXM102. TFEB activities and specific transcription factors for osteogenesis (Runx2) and adipogenesis (PPARγ) were also detected by immunofluorescent staining. Arrows indicate the sublocation of TFEB. **b** Quantitative gene analysis of Runx2 and PPARγ in **a**. **c** Representative images of aged hBMSCs treated with or without verteporfin (VP) and CXM102 in a bivalent differentiation system. Arrowheads indicated adipocytes stained with ARS. **d** Quantitative analysis of relative (i) mineralized nodule formation and lipid droplets in C. Results are presented as means ± SD, *n* = 3. *P < 0.05, **P < 0.01, n.s. means no significance

To explore the roles of TFEB in osteogenic and adipogenic commitment of hBMSCs regulated by CXM102, we induced aged hBMSCs to differentiation medium and detected the relationships between TFEB and specific transcription factors for osteogenesis and adipogenesis, Runx2 and PPARγ, respectively. When exposure to osteogenic induction, CXM102 increased osteogenesis of young hBMSCs revealed by ARS staining (Fig. 4a-i). At the molecular level, CXM102 promoted TFEB translocated into nucleus (Fig. S4a) and upregulated the gene and protein expression of Runx2 (Figs. 4b, S4b). However, VP inhibited the nuclear fraction of TFEB (Fig. S4a), downregulated both gene and protein levels of Runx2 (Figs. 4b, S4b) and decreased osteogenesis (Fig. 4a-i). Nevertheless, CXM102 could restore the VP-induced decline in osteogenesis. By contrast, CXM102 significantly induced TFEB translocation into nucleus (Fig. 4a-ii), but downregulated PPARγ expression (Figs. 4b and S4b) and inhibited adipogenesis of young hBMSCs while exposure to adipogenic induction (Fig. 4a-ii). Interestingly, VP also inhibited adipogenesis of young hBMSCs, and showed synergistic inhibition of adipogenesis with CXM102. These results suggested that CXM102 favored osteogenic differentiation of young hBMSCs.

The preferential shift of lineage commitment into adipocytes of aged BMSCs contributes to increased marrow adiposity and in turn impairs osteogenic regeneration and hematopoiesis. Recently, we have invented a new method that permitted both osteogenic and adipogenic differentiation of BMSCs in the same culture system.^50^ To better understand the effects of CXM102 on the fate determination of aged hBMSCs, we performed this bivalent differentiation assay to observed the final osteogenesis and adipogenesis. In control group, little mineralized nodules were formed as revealed by ARS staining, in which the lipid droplets were also distinguished (Fig. 4c, arrowheads). Meanwhile, more lipid droplets were detected by ORO staining (Fig. 4d), confirming the preferential differentiation into adipocytes of aged BMSCs. However, CXM102 remarkably reduced lipid droplets and increased mineralized nodules (Fig. 4c, d). Despite VP suppressed osteogenesis and promoted adipogenesis of aged hBMSCs, which was contrary to its effects on young hBMSCs, supplement of CXM102 reduced adipocytes and promoted osteogenesis. Furthermore, these results were further confirmed by gene and protein analysis of Runx2 and PPARγ (Fig. S4c, d), indicating that CXM102 could remarkably direct aged hBMSCs differentiation into osteoblasts at the expense of adipocytes. Taken together, these results suggested that CXM102 could remodel the bone-fat imbalance of aged hBMSCs in vitro.

### CXM102 promotes bone formation and ameliorates age-related bone loss in mice

**Fig. 5:**
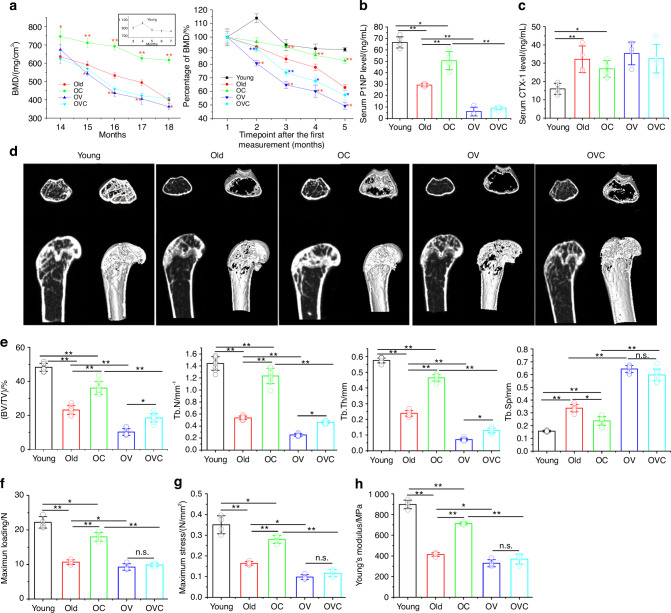
CXM102 ameliorates aged-related bone loss in male mice. **a** Representative curves of BMD value and the relative changes of BMD to the starting point followed by 4-month treatment with CXM102 (OC), verteporfin (OV), or both (OVC). Red asterisks indicate the comparing with the Old group, while blue asterisks indicate the comparing with OV group. Serum levels of P1NP (**b**) and CTX-1 (**c**) from 3-month (young), 14-month (old) and 18-month-old mice treated with CXM102 (OC), or verteporfin (OV), or both (OVC). **d** Representative 2D and 3D micro-CT images of the distal femoral trabecular bone microarchitecture. **e** Quantitative analysis of distal femoral parameters including bone volume to tissue volume (BV/TV), trabecular number (Tb. N), trabecular thickness (Tb. Th), and trabecular separation (Tb. Sp). Quantitative analysis of maximum loading (**f**) and maximum stiffiness (**g**) of femurs by a three-point bending assay. **h** Quantitative analysis of Young’s modulus of femurs. Results are presented as means ± SD, *n* ≥3. *P < 0.05, **P < 0.01

Considering the specifical osteogenesis of CXM102 controlled by VP, whether there were the same effects to regulate bone metabolism in vivo was still unknown. As bone mineral density (BMD) declined with ageing in all groups of mice, CXM102 significantly delayed the loss of BMD value compared with other groups, except for the young mice (Fig. 5a). After a 4-month-long treatment with the drugs, serum levels of the bone formation marker (N-terminal propeptide of type I procollagen, PINP) and bone resorption marker (C-telopeptide of type I collagen, CTX-I) were also analyzed. Compared with the 3-month-old mice (Young), serum PINP was remarkedly decreased in old mice, but was significantly upregulated by administration of CXM102 (Fig. 5b). Conversely, serum CTX-1 increased with aging, but didn’t significantly change upon CXM102 stimulation (Fig. 5c), suggesting that CXM102 may mainly regulate bone formation in vivo.

Subsequently, we assessed the effects of CXM102 on bone microarchitecture of the distal femurs by micro-CT scanning. As shown in 2D and 3D images, CXM102 significantly reduced total bone loss compared with the old mice (Fig. 5d, e). Meanwhile, a significant increase of total bone volume (BV/TV), number (Tb. N) and thickness (Tb. Th) of trabecular bone, as well as a decrease of trabecular separation (Tb.Sp.) were presented in OC mice compared with the old mice (Fig. 5e). Furthermore, the maximum loading of bone fracture (Fig. 5f), stress (Fig. 5g) and Young’s modulus (Fig. 5h) in CXM102-treated mice were also significantly increased compared with the old mice, suggesting that CXM102 could effectively improve bone strength and prevent fragility fracture of senile osteoporosis.

However, pretreatment with VP severely attenuated the bone anabolic effects of CXM102 in vivo, leading to lower BMD and serum P1NP, less bone mass and mechanical properties (Fig. 5). In addition, it was also important to note that long-term injection of VP may accelerate the bone loss and worsened senile osteoporosis, but the mechanisms remained unclear.

### CXM102 remodels the bone-fat imbalance of aged marrow stromal cells in aged mice

**Fig. 6:**
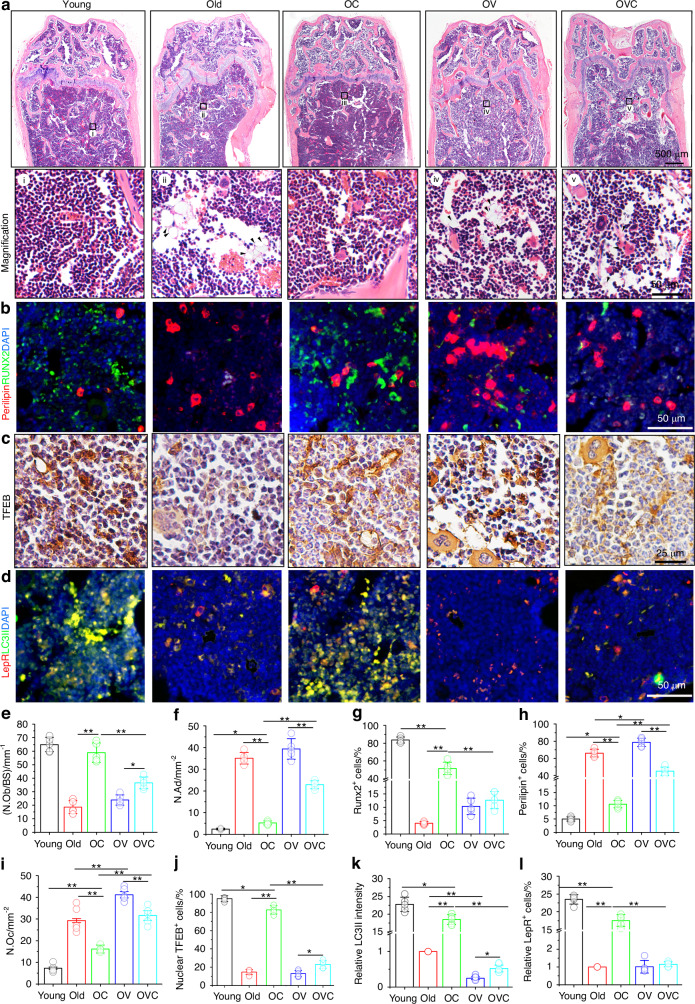
CXM102 remodels the bone-fat imbalance of aged marrow stromal cells in middle aged mice. **a** Representative images of H&E staining for histological analysis of distal femurs from the indicated mice. (i-v) Enlarged areas from each section. **b** Double-staining of perilipin and Runx2 in bone marrow sections. **c** Immunohistologic analysis of TFEB in marrow cells. **d** Immunofluorescent double-staining of LepR and LC3 in bone marrow sections. Quantitative analysis of osteoblasts (**e**), adipocytes (**f**), Runx2 positive cells (**g**), perilipin positive cells (**h**), osteoclasts (**i**), nuclear TFEB positive cells (**j**), relative LepR positive cells (**k**) and LC3II intensity (**l**) to old mice. Results are presented as means ± SD, *n* = 5. *P < 0.05, **P < 0.01

Aged-related osteoporosis is featured with low bone mass and excessive accumulation of adipocytes in bone marrow. Whether the bone anabolic effects of CXM102 under aging were attributed to the lineage shift of aged BMSCs remained unclear. Next, we focused on the effects of CXM102 on bone marrow cells. In contrary to young mice, there were more adipocytes (Fig. 6a-ii, arrowheads) and less osteoblasts in old marrow, but CXM102 administration significantly increased osteoblasts and reduced adipocytes accumulation (Fig. 6a, e, f). These results were further confirmed by specific staining of Runx2 and perilipin (Fig. 6b, g, h). Then we immediately analyzed the effects of CXM102 on TFEB expression of bone marrow cells. Consistently, ageing led to decreased the nuclear expression of TFEB in marrow cells, while CXM102 dramatically increased the nuclear fraction in old mice (Fig. 6c, j). Meanwhile, CXM102 also elevated Runx2 level in marrow cells as revealed by colocalization staining with TFEB (Fig. S4a, c), suggesting the enhanced nuclear level of TFEB triggered by CXM102 promoted osteogenesis in old mice. Similar to the aged hBMSCs, pretreatment with VP blocked the bone-forming effects of CXM102 and reduced the nuclear TFEB positive stromal cells.

Recent studies have demonstrated that BMSCs expressing leptin receptor (LepR^+^) were major source of bone and marrow adipocytes.^24,51,52^ To investigate if CXM102 could enhance the autophagy activities of LepR^+^ BMSCs in vivo, we examined LepR and LC3II expression in the trabecular bone area of distal femur. Our results revealed that the double-positive cells in OC mice were significantly outnumbered by the old group, but was distinctly diminished by VP (Fig. 6d, k, l). These results indicated that CXM102 could promote autophagy activities of LepR^+^ BMSCs in vivo, but was also inhibited by VP.

Notably, myelofibrosis was also observed in bone marrow of old mice (Fig. S5b, green arrowheads), and VP treatment exacerbated the myelofibrosis. To our surprise, CXM102 significantly suppressed the myelofibrosis in old mice (Fig. S5b, d). Consequently, these results indicated that CXM102 was not only able to remold bone-fat imbalance but also create a healthier environment for fate choice of aged marrow cells under aging.

### CXM102 reduces serum inflammation and increases healthspan and longevity in male mice

**Fig. 7:**
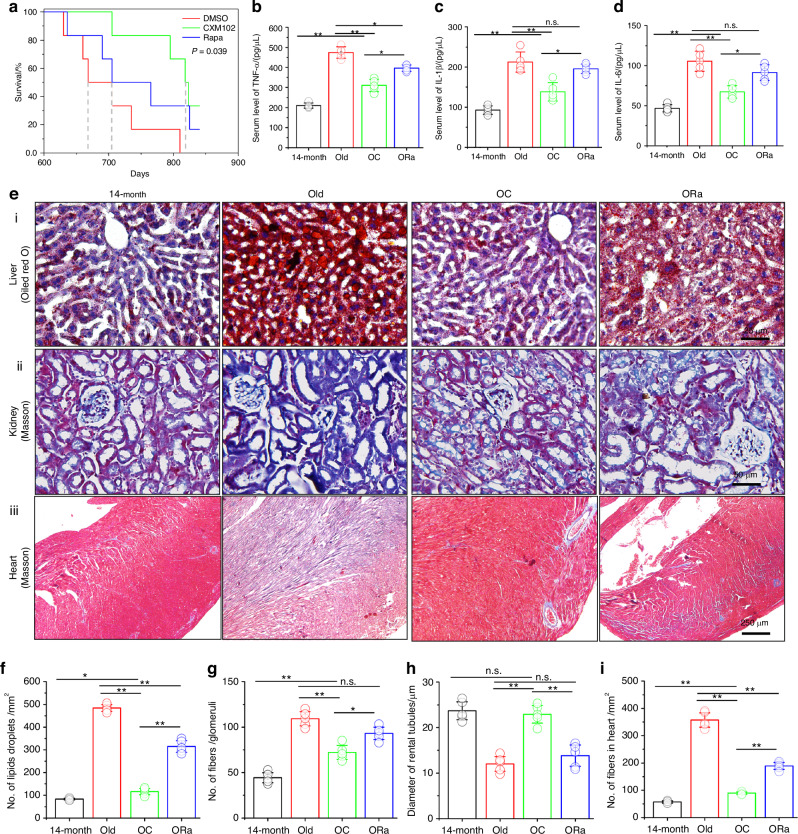
Long-term treatment with CXM102 increases healthspan and lifespan in male mice. **a** Kaplan–Meier survival curve for mice treated either with DMSO, or rapamycin, or CXM102. Gray dashed lines indicated median lifespan. Statistic analysis of serum levels of TNF-α (**b**), IL-1β (**c**) and IL-6 (**d**) at 14-month (before injection) and 20-month (6-month after injection with DMSO, or rapamycin, or CXM102). **e** Representative images of histological analysis from liver (i, Oiled red O staining), kidney (ii, Masson’s staining) and heart (iii, Masson’s staining). Quantitative analysis of lipid droplets in liver (**f**), numbers of fibers in glomeruli (**g**) and diameter of renal tubules (**h**) in kidney and numbers of fibers (**i**) in heart. Results are presented as means ± SD, *n* = 5. *P < 0.05, **P < 0.01, n.s. means no significance

Given that rejuvenation of BMSCs and autophagic promotion have shown great benefits for decreased inflammation and healthspan improvement, we next determined the long-term effects of CXM102 starting at middle age (14-month-old) in male C57BL/6 mice on aged-related inflammation and lifespan. As low-dose (8 mg/kg weight) of rapamycin is considered as gold standard for healthy aging,^34^ we compared the long-term effects of the same dose of CXM102 on survival and health parameters with rapamycin (Rapa). The survival curves of control (DMSO), CXM102- and Rapa-treated mice began to separate at 3 months of administration (Fig. 7a). While rapamycin injection led to a 5.68% extension of median lifespan, CXM102 increased up to 22.3%. Chronic inflammation has been considered as a hallmark of aging. In this study, the serum levels of TNF-α, IL-1β and IL-6 in CXM102-treated mice were significantly lower than old and rapamycin-treated mice (Fig. 7b–d). However, our work also showed that rapamycin failed to reduce serum levels of IL-1β and IL-6 compared with control mice (Fig. 7c, d), despite it diminished serum level of TNF-α (Fig. 7b). This result suggested that CXM102 may had a better effect on inflammaging than rapamycin.

Aging is also characterized by multi-organ dysfunction, including but not limited to non-alcoholic fatty liver disease (NAFLD), renal fibrosis and myocardial fibrosis, and rapamycin has been shown to extend lifespan and healthspan effectively.^34,35^ In this work, we meanwhile compared the histomorphology changes of these main organs treated with DMSO, CXM102 or rapamycin. As fat droplets occurred in liver with aging, 6-month treatment with CXM102 diminished a larger number of fat accumulation than rapamycin did (Fig. 7e–i, f). While rapamycin had little and no significant impacts on renal fibrosis and diameter of renal tubules, CXM102 largely reduced renal fibrosis and increased the diameter of renal tubules, respectively (Fig. 7e-ii, g, h). In parallel to the changes occurred in kidney, heart fibrosis was also substantially decreased in CXM102-treated mice (Fig. 7e-iii and l). Taken together, these results suggested the more beneficial effects of CXM102 on healthy aging than rapamycin.

## Discussion

Autophagy plays fundamental roles in the homeostasis and fate determination of BMSCs,^15^ and an age-related decline in autophagy is widely accepted as one hallmark of aging.^25,27,53^ Impaired autophagy resulted in adipogenic differentiation,^54^ while increased autophagy led to osteogenic differentiation of BMSCs^32^ and improve the therapeutic application of BMSCs.^55^ In this study, we provided a novel autophagy inducer, CXM102, showing better benefits than rapamycin, to rejuvenate aged hBMSCs through the enhanced nuclear translocation of TFEB. It is noteworthy that CXM102 had no effects on normal cell growth, but exhibited inhibitory effects on multiple cancer cells (Fig. 1d). As autophagy may act as a double-edged sword that both helps tissue homeostasis and facilitates cancer development,^28,56^ further studies are required to explore the unknown roles of CXM102 in cancers.

TFEB is a master transcriptional regulator of lysosomal biogenesis and autophagy, but little is known about the relationship between TFEB activities and fate decision of aged hBMSCs. In this work, we found that total protein level of TFEB and the targeting genes were downregulated by siTFEB within 24 h, but restored to the initial levels at 72 h after transfection (Fig. S3a, b). This phenomenon could be explained by the immediate disruption of total *tfeb* transcription caused by siTFEB transfection, thus leading to downregulated TFEB level. But when cultured cells were proliferating, the dose of siTFEB was not enough to disrupt the RNA strands. Considering autophagy is an ongoing and dynamic process, it was not suitable for siRNA to perform long term experiments like differentiation. Fortunately, we found VP had a specific role in prevention of TFEB nuclear translocation (Fig. 3c). VP is an FDA-approved drug that normally used for photodynamic therapy in clinical, but it also inhibits autophagy without light activation.^41^ Nevertheless, little is known about the detailed effects involved in autophagy. Here we demonstrated that VP could inhibit nuclear translocation of TFEB to suppress the formation of autophagolysosome, decrease osteogenesis in aged hBMSCs. Interestingly, VP has been shown to attenuate heterotopic ossification by inhibiting osteogenesis of tendon stem cells via YAP/β-catenin pathway.^57^ Consistent with 3-MA (Fig. S2), VP also inhibited the cytoprotective effects of CXM102 on senescence of hBMSCs (Fig. S3d–f). Recent studies showed that the transcriptional activities of TFEB were mainly controlled by mTORC1 and AMPK kinases,^37,38,49,58^ but how CXM102 regulated the intracellular sublocation of TFEB is still unclear.

Another hallmark of aged BMSCs is the skewed differentiation potency from osteoblast to adipocyte,^7^ featured by increased lipid droplets formation and less mineralized nodules when exposure to differentiation medium. We found that CXM102 not only enhanced the nuclear translocation of TFEB, but also promoted Runx2 expression in nucleus in respond to osteogenic induction (Fig. 4a-i). Hence, there may be a synergetic effect of CXM102 with Runx2 to regulate the osteogenic differentiation of BMSCs. By contrast, CXM102 specifically inhibited PPARγ expression, thus leading to less lipid droplets formation (Fig. 4a-ii). Additionally, we conducted a bivalent differentiation system to observe the effects of CXM102 on lineage changes of aged hBMSCs. Our results indicated that CXM102 remarkably promoted osteogenesis and suppress adipogenesis of aged BMSCs (Fig. 4c, d), indicating the powerful bone-forming effects of CXM102 that directed osteogenic differentiation of aged BMSCs.

SOP is featured with increased marrow adipocytes, decreased BMD, bone mass and strength, leading to an increased risk of fractures.^59^ Here we demonstrated that CXM102 significantly delayed the loss of BMD and bone mass, but augmented bone strength (Fig. 5), suggesting its potentially use for osteoporosis. As the only tissue where bone and fat coexist in the same microenvironment, the bone marrow offers a unique window into the investigation of molecular events governing the lineage commitment of BMSCs.^6^ Considering that CXM102 promoted the nuclear translocation of TFEB, autophagy and preferential osteogenesis of aged hBMSCs in vitro, the in vivo anti-osteoporosis effects could be reasonably explained by the lineage shift of aged BMSCs conducted by CXM102, which was consistent with reduced marrow adipocytes, increased osteoblasts and autophagy activities (Fig. 6).

Besides, myelofibrosis is another challenge of aging. We found that myelofibrosis increased with age, but CXM102 significantly inhibited the myelofibrosis (Fig. S4b). Since LepR^+^ BMSCs have been identified as key components of hematopoietic microenvironment^52,60,61^ and responsible for myelofibrosis,^62,63^ it was reasonable that enhancement of TFEB-mediated autophagy by CXM102 treatment in LepR^+^ BMSCs could reduce the myelofibrosis, but further studies are needed to illustrate the detailed mechanisms.

In addition, we also found that long-term injection of VP accelerated bone loss and weaken the strength, accompanied with less osteoblasts, more adipocytes and osteoclasts, reduced nuclear TFEB activities in marrow cells, reminding that more attention should be paid for clinical use, despite CXM102 could partially alleviate the adverse effects (Figs. 5 and 6). Taken together, these results suggested that CXM102 could remodel a healthier osteogenic microenvironment in aged bone marrow for lineage allocation of BMSCs and hematopoiesis in vivo.^64^

Aging is characterized by accumulation of chronic inflammation and progressive loss of physiological integrity, leading to multiorgan dysfunction and reduced quality of life.^18,65–67^ Accumulation of marrow adipocytes results in increased inflammation, and osteoclast formation and bone resorption by secreting (pro)inflammatory factors. Compared with control and rapamycin-treated mice, our results revealed that CXM102 significantly reduced serum levels of (pro)inflammatory factors, including TNF-α, IL-1β and IL-6 (Fig. 7b–d). Given that bone remodeling plays key roles in homeostasis of bone marrow microenvironment,^64^ we proposed that the increased bone anabolism and attenuated serum inflammation may contribute to healthier hematopoiesis for bone and other organs that led to better survival in CXM102-treated mice. Osteoimmunology is termed to study the interactions between skeletal and immune systems,^68–70^ but whether CXM102 play roles in marrow inflammation and immune development is of great interest in future studies.

In summary, we clearly demonstrated the novel compound CXM102 could rejuvenate and remodel bone-fat imbalance of aged BMSCs via enhanced nuclear translocation of TFEB, leading to alleviated bone loss and increased healthspan and longevity in middle age male mice. Our results will shed new lights on therapeutic strategies for senile osteoporosis and healthy aging.

## Limitation of this study

We acknowledge several limitations of this study. First, we did not determine the effects of CXM102 on female mice, mainly because of the possible perturbation of estrogen deficiency in female mice for senile osteoporosis. Second, only 4 and 5 mice were analyzed in the study of osteoporotic features and longevity, respectively. Despite the data within each group are relatively centralized, larger number of both male and female mice will be required to confirm the anti-osteoporosis effects of CXM102 in vivo. Third, whether the LepR^+^ BMSCs are truly responsive to CXM102 and how the lineage choice changes in mice are unclear. Maybe more sophisticated experimental techniques like lineage-tracing models, will address these urgent issues. Finally, the post-transcriptional modification of TFEB has been extensively elucidated, yet it is important to uncover the mechanism that how CXM102 regulates the TFEB activities in subsequent studies.

## Materials and methods

### Materials and reagents

All reagents used for cell culture, including low-glucose Dulbecco’s Modified Eagle’s Media (DMEM; Gibco, 11054, 020), fetal bovine serum (FBS; Gibco, 10099, 141C), non-essential amino acids (Gibco, 11140, 050), antibiotics (P/S; Gibco, 15070, 063) and trypsin (Gibco, 15400, 054) were purchased from Thermo Fisher Scientific Incorporation. Recombinant basic fibroblasts growth factor (bFGF; 100-18B), epidermal growth factor (EGF; AF-100-15) were purchased from PeproTech Inc.. PBS (P1020), Chloroquine (CQ; IC4440), rapamycin (R8140) and 3-Methyladenine (3-MA; IM0190) were purchased from Beijing Solarbio Science & Technology Co., Ltd (Solarbio Inc.). Hydrogen peroxide solution (H_2_O_2_; Sigma, 88597) was from Merck group. All materials and regents for western blotting were purchased from Beyotime Institute of Biotechnology (Beyotime), including polyvinylidene difluoride membranes (FFP19), Bovine Serum Albumin (BSA; ST023), TBST buffer (ST673).

All drugs used for in vitro differentiation of hBMSCs, including dexamethasone (Sigma; D4902), β-glycerophosphate (Sigma; G9422), L-ascorbic acid (Sigma; A4544), indomethacin (Sigma; I7378), isobutyl methylxanthine (Sigma; IBMX, I7018) were purchased from Merck. Insulin (I8830) was from Solarbio Inc. Verteporfin was from MedChemExpress LLC (MCE; CL318952). CXM102 was synthesized in our lab as described in a patent (CN23NN14118I).

Primary and secondary antibodies used in this study were shown in Table S1. Cytochemical staining dyes, including Alizarin red S (G8550) and Oil red O (O8010) were from Solarbio Inc. The working solution was prepared based on the manufacture’s instruction.

### Animal approval

All animal experiments were performed in accordance with the protocol approved by Guangxi University of Chinese Medicine Institutional Welfare and Ethical Committee (Approval No. DW2022). C57/BL6 mice were reared in a pathogen-free facility at a controlled temperature (23–25 °C), under a 12 h light and dark cycle with food and water provided *ad libitum*. The cage and bedding were changed once a week. 3-month-old and 14-month-old mice were used in this study.

### Cell culture

Young, old and immortal hBMSCs, normal synoviocytes, FEK4 and RA-FLS were kindly gift from Dr. Li Yang (Chongqing University) and maintained as described before.^50,71^ Human normal liver cells (L02) and cancer cell lines, including Hep3B, HCCLM3, HCT116, DU145, A549 and THP-1 were from Dr. Erwei Hao (Guangxi University of Chinese Medicine). Briefly, 5 × 10^5^ cells/mL were plated in a 100-mm diameter cell culture dish containing low-glucose DMEM supplemented with 10% FBS, 100 U/mL P/S, 0.1 mmol/L non-essential amino acids, 20 ng/mL bFGF and 20 ng/mL EGF. Cells were maintained in a humidified incubator at 37 °C with 5% CO_2_. When 70% to 80% confluent, adherent cells were trypsinized with 0.05% trypsin-1mmol/L EDTA at 37 °C for 2 min, harvested, and expanded for future use.

### Effects of CXM102 on cell growth

To study the effects of CXM102 on cell growth, a density of 2000 cells per well of indicated cell types were seeded into a 96-well plate as described before.^71^ cells were treated with different concentrations of CXM102 (0, 5, 10, 20, 30, 50 μmol/L) for different time points (12, 24 or 36 h). Cell activities were determined by Cell Counting Kit-8 kit (Beyotime, C0038) following the manufacturer’s instruction.

### Effects of CXM102 on hBMSCs senescence

To study the effects of CXM102 on cellular senescence, hBMSCs were pretreated with different concentrations of CXM102 (0, 5, 10, 20 μmol/L) 2 h before 200 μmol/L H_2_O_2_ addition into culture medium for 2 h at 37 °C, as described by others,^72^ followed by fresh medium change containing 20 μmol/L H_2_O_2_ for 4 h. Then hBMSCs were fixed and analyzed the levels of senescence β-Galactosidase activities (SA-β-Gal; Beyotime, C0602), reactive oxygen species (ROS; Beyotime, S0033S), mitochondrial membrane potential (MMP; JC-1, Beyotime, C2006) according to the manufacturer’s instruction. Molecules levels of p16INK4a and γH2AX, osteogenic and adipogenic differentiation were also detected as described below.

### Effects of CXM102 on autophagy

To investigate the roles of autophagy and transcription factor EB (TFEB) in hBMSCs senescence, 2.5 mmol/L 3-MA or 50 μmol/L CQ was added into the culture medium for 4 h before CXM102 treatment. Then cells were fixed and detected by immunofluorescence (IF) or western blotting for the analysis of autophagic influx, autophagosomes, autolysosomes and TFEB activities as described below.

### Immunofluorescent (IF) /Immunohistological (IHC) analysis

For immunofluorescent/immunohistochemical staining, cells or deparaffined sections of femurs were fixed with 4% paraformaldehyde (Beyotime, P0099), permeatilized with 0.2% Triton X-100 (Solarbio, T8200), and blocked with 1% bovine serum albumin (Solarbio, SW3015). Then samples were incubated with primary antibodes (Table S1) or non-immune antiserum as a negative control at 4°C overnight. After washing with PBS, sections were incubated with HRP-conjugated or fluorescent secondary antibodies (Table S1) for 1 h, and counterstained with DAPI for 10 min at room temperature. Images were obtained by upright microscope (BX53, Olympus), or fluorescence microscopy (DMI8, Leica). For quantitative analysis, at least four independent samples of each group were analyzed by Image-Pro Plus 6.0 software.

### RNA interference and transfection

To study the role of TFEB in CXM102-mediated autophagy, the following sequence of siRNA was used for TFEB downregulation. siRNA scramble sense, 5’-UAGCGACUAAACACAUCAAU‑3’ and antisense, 5’‑UUGAUGUGUUAGUCGCUAU‑3’; siTFEB sense, 5’-GGAUCAAGGAGCUGGGAAUUU‑3’ and antisense, 5’- AUUCCCAGCUCCUUGAUCCUU-3’ (GenePharma, Suzhou, China). The transfection of siRNA was conducted as we did before.^73^ Briefly, hBMSCs (2 × 10^5^ cells per well) were seeded into a 24-well plate, then 50 nmol/L siRNA scramble or siTFEB was transfected into hBMSCs with Lipofectamine 2000 (Invitrogen, 11668, 019) according to the manufacturer’s instructions. Finally, the protein level was assessed by IF staining or western blotting.

### Quantitative real-time PCR

Total cellular RNAs were isolated using RNA Extraction Kits (Solarbio, R1200) according to the manufacturer’s instructions and quantified by Nanodrop One C (Thermo Fisher Scientific, Madison, USA). 1 μg RNA was analyzed by using a TaqMan One Step RT-qPCR Kit (Solarbio, T2210) on LightCycle 96 Instrument (Roche, Mannhein, Germany) according to the manufacturer’s instructions. The primers used in this study are shown in Table S2. The relative expression of target genes was normalized to *Gapdh* and calculated using the 2^−∆∆CT^ method.

### Western blotting

Total protein was extracted using a protein extraction kit (Solarbio, BC3710) and the protein concentration was determined by BCA (Beyotime, P0012) assay according to the manufacturer’s instructions. Next, 25 μg of protein sample was subjected to 12% sodium dodecyl sulfate-polyacrylamide gel electrophoresis, followed by transfer to polyvinylidene difluoride membranes. Then the membranes were blocked with 3% BSA in TBST buffer for 2 h at room temperature, followed by incubation with the indicated primary antibodies (Table S1) at 4 °C overnight and horseradish peroxidase-conjugated secondary antibodies for 1 h at room temperature. Targeted proteins were visualized with enhanced chemiluminescence solution (Beyotime, P0018S) and detected using a ChemiDoc Touch Imaging system (Bio-Rad, CA, USA).

### Effects of CXM102 on differentiation induction

Osteogenic, adipogenic, and bivalent differentiation and detection of BMSCs were strictly conducted as we described in a recent method article.^50^

### Animal experiments

To study the effects of CXM102 on aged-related bone metabolism, 14-month-old mice were divided into four groups, followed by a period of 4-month-long treatment with DMSO (Indicated as “Old”), CXM102 alone (OC; 20 mg/kg body weight), verteporfin alone (OV; 10 mg/kg) and CXM102 combined with verteporfin (OVC). In OVC group, verteporfin was pretreated 2 weeks before CXM102 injection. All drugs were dissolved into 50 μL DMSO and injected subcutaneously twice a week. Mice were sacrificed 1 month after the final injection, serum and femurs were collected for analysis.

### BMD measurement

To assess the effects of CXM102 on BMD changes in vivo, the BMD values of 14-month-old mice were measured as the initial time and then followed by a period of 4-month injection with DMSO (Marked as “Old”), CXM102 (OC), verteporfin (OV) alone or both (OVC). In addition, 3-month-old untreated mice (Marked as “Young”) were used as positive control in this experiment. BMD was measured once a month by dual energy x-ray absorptiometry (DXA) as described by others.^74^ Briefly, mice were anesthetized with isoflurane (Yuyan Corporation, Shanghai, China) in a chamber, then were placed on the Lunar PIXImus densitometer platform (GE Medical-Lunar, Madison, WI, USA). Half of the distal femur was selected as area of interest and perform a scout scan. BMD value was recorded by the PIXImus software (Lunar Piximus 2.0).

### ELISA assay

Mouse blood serum was diluted 1:1 using PBS and the diluted serum levels of P1NP (Sangon Biotech, D721053), CTX-1; (Sangon Biotech, D721204), TNF-α (Sangon Biotech, D721217), IL-1β (Sangon Biotech, D721017) and IL-6 (Sangon Biotech, D721022) were measured by ELISA kits according to the manufacture’s instruction.

### MicroCT analysis

MicroCT analysis was performed using the settings according to the manufacture’s instruction. Generally, Mouse femurs were dissected, fixed with 4% paraformaldehyde (Beyotime, P0099) for 48 h and stored in 70% ethanol, loaded into 10-mm diameter scanning tubes, and scanned with a peak tube voltage of 50 kV and current of 0.145 mA at 18 μm resolution, (Latheta LCT200, Hitachi Aloka, Japan). The 2D and 3D model visualization software (BeeViewer v3.4.1) and data analysis software (VGStudioMAX v2.2) were applied to analyze the distal femoral metaphyseal trabecular bone and the parameters of the diaphyseal cortical bone. We defined a region of interest (ROI) as the area between 2%–6% proximal to the growth plate in the distal femora. Cross-sectional images of the femora were established for 3D histomorphometry analysis of the trabecular bone. The percentage of bone volume (BV/TV), the number (Tb. N), thickness (Tb. Th) and space (Tb. Sp) of trabecular bone were analyzed.

### Histological analysis

Histological analysis of femurs was performed as we did before.^71^ Briefly, the dissected distal femurs were fixed in 4% paraformaldehyde (Beyotime, P0099) for 48 h, decalcified with 15% EDTA (pH7.2-7.4) and were paraffin embedded. 5mm-thickness sections were cut and subjected to haematoxylin and eosin (H&E; Solarbio, G1120) or Masson’s trichrome staining (Solarbio, G1340). Images were taken by a microscope (BX53, Olympus) and analyzed by Image-Pro Plus 6.0 (Media Cybernetics, MD, USA) software.

### Biomechanical testing

To investigate mechanical strength of femurs, an electronic universal material testing machine (AGS-10kNJ, Shimadzu, Japan) was used for the three-point bending test as described in other’s experiment.^31^ Before the test, the long and short diameters of the femoral shaft were measured with a Vernier caliper, then femurs were placed between two support abutments. The load was applied to middle of the femoral shaft through a touching probe. During this test, the posterior surface was in tension, and the anterior surface was in compression. The strength variables (bending modulus of elasticity, bending energy, maximum bending stress, and bending rigidity coefficient) were calculated with a standard engineering formula.

### Lifespan analysis

To compared the effects of CXM102 and rapamycin on lifespan, middle-aged mice were divided into three groups, which were administered with rapamycin (8 mg/kg weight), CXM102 (8 mg/kg weight) or DMSO twice a week for a period of 6-month-long term. 1 month after the final injection, three mice from each group were sacrificed for assessment of histological changes of liver, kidney and heart. The other mice were freely fed until natural death. Survival curves were calculated by Origin 8.0 (OriginLab, Guangzhou, China).

### Statistical analysis

Results are represented as means ± standard deviations. Statistical analysis was performed using Student’s t-test as well as one-way analysis of variance (ANOVA) followed by the Tukey HSD test for post hoc comparison (Origin 8.0, OriginLab). Difference was considered significant when *P* < 0.05 indicated as *, while more significant when *P* < 0.01 indicated as **.

### Supplementary information

supplement data-clean version

## Data Availability

All data generated in this study are available from the corresponding author upon request.

## References

[1] Krampera, M. & Le Blanc, K. Mesenchymal stromal cells: putative microenvironmental modulators become cell therapy. *Cell Stem Cell* **28**, 1708–1725 (2021). 34624232 10.1016/j.stem.2021.09.006

[2] Zupan, J. et al. Age-related alterations and senescence of mesenchymal stromal cells: Implications for regenerative treatments of bones and joints. *Mech. Ageing Dev.* **198**, 111539 (2021). 34242668 10.1016/j.mad.2021.111539

[3] Cheng, M., Yuan, W., Moshaverinia, A. & Yu, B. Rejuvenation of mesenchymal stem cells to ameliorate skeletal aging. *Cells* **12**, 998 (2023). 37048071 10.3390/cells12070998 PMC10093211

[4] Qadir, A. et al. Senile Osteoporosis: the involvement of differentiation and senescence of bone marrow stromal cells. *Int. J. Mol. Sci.* **21**, 349 (2020). 31948061 10.3390/ijms21010349 PMC6981793

[5] Liu, Z. Z. et al. Autophagy receptor OPTN (optineurin) regulates mesenchymal stem cell fate and bone-fat balance during aging by clearing FABP3. *Autophagy* **17**, 2766–2782 (2021). 33143524 10.1080/15548627.2020.1839286 PMC8526045

[6] Yu, B. et al. PGC-1alpha controls skeletal stem cell fate and bone-fat balance in osteoporosis and skeletal aging by inducing TAZ. *Cell Stem Cell* **23**, 193–209.e195 (2018). 30017591 10.1016/j.stem.2018.06.009 PMC6322535

[7] Li, H. et al. FOXP1 controls mesenchymal stem cell commitment and senescence during skeletal aging. *J. Clin. Invest.* **127**, 1241–1253 (2017). 28240601 10.1172/JCI89511 PMC5373872

[8] Wang, Y. et al. Alpha-ketoglutarate ameliorates age-related osteoporosis via regulating histone methylations. *Nat. Commun.* **11**, 5596 (2020). 33154378 10.1038/s41467-020-19360-1 PMC7645772

[9] Picke, A. K. et al. Thy-1 (CD90) promotes bone formation and protects against obesity. *Sci. Transl. Med.* **10**, eaao6806 (2018). 10.1126/scitranslmed.aao6806 30089635

[10] Deng, P. et al. Loss of KDM4B exacerbates bone-fat imbalance and mesenchymal stromal cell exhaustion in skeletal aging. *Cell Stem Cell* **28**, 1057–1073.e1057 (2021). 33571444 10.1016/j.stem.2021.01.010 PMC8178178

[11] Ye, L. et al. Histone demethylases KDM4B and KDM6B promotes osteogenic differentiation of human MSCs. *Cell Stem Cell* **11**, 50–61 (2012). 22770241 10.1016/j.stem.2012.04.009 PMC3392612

[12] Cai, G. P. et al. Alkbh1-mediated DNA N6-methyladenine modification regulates bone marrow mesenchymal stem cell fate during skeletal aging. *Cell Prolif.* **55**, e13178 (2022). 35018683 10.1111/cpr.13178 PMC8828262

[13] Xiao, Y. et al. Splicing factor YBX1 regulates bone marrow stromal cell fate during aging. *EMBO J.* **9**, e111762 (2023). 10.15252/embj.2022111762 PMC10152142 36943004

[14] Wu, Y. et al. Mettl3-mediated m6A RNA methylation regulates the fate of bone marrow mesenchymal stem cells and osteoporosis. *Nat. Commun.* **9**, 4772 (2018). 10.1038/s41467-018-06898-4 PMC6235890 30429466

[15] Chen, X. D. et al. Autophagy in fate determination of mesenchymal stem cells and bone remodeling. *World J. Stem Cells* **12**, 776–786 (2020). 32952858 10.4252/wjsc.v12.i8.776 PMC7477662

[16] Wang, J. et al. The role of autophagy in bone metabolism and clinical significance. *Autophagy* **19**, 2409–2427 (2023). 10.1080/15548627.2023.2186112 PMC10392742 36858962

[17] Yang, R. et al. Premature aging of skeletal stem/progenitor cells rather than osteoblasts causes bone loss with decreased mechanosensation. *Bone Res.* **11**, 35 (2023). 37407584 10.1038/s41413-023-00269-6 PMC10322990

[18] Cai, Y. et al. The landscape of aging. *Sci. China Life Sci.* **65**, 2354–2454 (2022). 36066811 10.1007/s11427-022-2161-3 PMC9446657

[19] Liu, H. et al. PTH regulates osteogenesis and suppresses adipogenesis through Zfp467 in a feed-forward, PTH1R-cyclic AMP-dependent manner. *Elife* **12**, e83345 (2023). 10.7554/eLife.83345 PMC10171860 37159501

[20] Zhang, J. et al. The effect of parathyroid hormone on osteogenesis is mediated partly by osteolectin. *Proc. Natl. Acad. Sci. USA* **118**, e2026176118 (2021). 10.1073/pnas.2026176118 PMC8237660 34140410

[21] Wu, H., Xue, Y., Zhang, Y., Wang, Y. & Hou, J. PTH1-34 promotes osteoblast formation through Beclin1-dependent autophagic activation. *J. Bone Miner. Metab.* **39**, 572–582 (2021). 33818629 10.1007/s00774-021-01212-7

[22] Fan, Y. et al. Parathyroid hormone directs bone marrow mesenchymal cell fate. *Cell Metab.* **25**, 661–672 (2017). 28162969 10.1016/j.cmet.2017.01.001 PMC5342925

[23] Wein, M. N. & Kronenberg, H. M. Regulation of bone remodeling by parathyroid hormone. *Cold Spring Harb. Perspect. Med.* **8**, a031237 (2018). 10.1101/cshperspect.a031237 PMC6071549 29358318

[24] Yang, M. et al. Parathyroid hormone shifts cell fate of a leptin receptor-marked stromal population from adipogenic to osteoblastic lineage. *J. Bone Min. Res.* **34**, 1952–1963 (2019). 10.1002/jbmr.3811 31173642

[25] Aman, Y. et al. Autophagy in healthy aging and disease. *Nat. Aging* **1**, 634–650 (2021). 34901876 10.1038/s43587-021-00098-4 PMC8659158

[26] Yin, X. et al. Autophagy in bone homeostasis and the onset of osteoporosis. *Bone Res.* **7**, 28 (2019). 10.1038/s41413-019-0058-7 PMC6804951 31666998

[27] Lopez-Otin, C., Blasco, M. A., Partridge, L., Serrano, M. & Kroemer, G. Hallmarks of aging: an expanding universe. *Cell* **186**, 243–278 (2023). 36599349 10.1016/j.cell.2022.11.001

[28] Mizushima, N. & Levine, B. Autophagy in human diseases. *N. Engl. J. Med*. **383**, 1564–1576 (2020). 33053285 10.1056/NEJMra2022774

[29] Li, X. et al. Targeting autophagy in osteoporosis: from pathophysiology to potential therapy. *Ageing Res. Rev.* **62**, 101098 (2020). 32535273 10.1016/j.arr.2020.101098

[30] Onal, M. et al. Suppression of autophagy in osteocytes mimics skeletal aging. *J. Biol. Chem.* **288**, 17432–17440 (2013). 23645674 10.1074/jbc.M112.444190 PMC3682543

[31] Wu, J. et al. Rapamycin improves bone mass in high-turnover osteoporosis with iron accumulation through positive effects on osteogenesis and angiogenesis. *Bone* **121**, 16–28 (2019). 30610968 10.1016/j.bone.2018.12.019

[32] Ma, Y. et al. Autophagy controls mesenchymal stem cell properties and senescence during bone aging. *Aging Cell* **17**, e12709 (2018). 29210174 10.1111/acel.12709 PMC5770781

[33] Partridge, L., Fuentealba, M. & Kennedy, B. K. The quest to slow ageing through drug discovery. *Nat. Rev. Drug Discov.* **19**, 513–532 (2020). 32467649 10.1038/s41573-020-0067-7

[34] Zhang, Y., Zhang, J. & Wang, S. The role of rapamycin in healthspan extension via the delay of organ aging. *Ageing Res. Rev.* **70**, 101376 (2021). 34089901 10.1016/j.arr.2021.101376

[35] Sharp, Z. D. & Strong, R. Rapamycin, the only drug that has been consistently demonstrated to increase mammalian longevity. An update. *Exp. Gerontol.* **176**, 112166 (2023). 37011714 10.1016/j.exger.2023.112166 PMC10868408

[36] Mannick, J. B. & Lamming, D. W. Targeting the biology of aging with mTOR inhibitors. *Nat. Aging*, **3**, 642–660 (2023). 10.1038/s43587-023-00416-y PMC10330278 37142830

[37] Abokyi, S., Ghartey-Kwansah, G. & Tse, D. Y. TFEB is a central regulator of the aging process and age-related diseases. *Ageing Res. Rev.* **89**, 101985 (2023). 10.1016/j.arr.2023.101985 37321382

[38] Tan, A., Prasad, R., Lee, C. & Jho, E. H. Past, present, and future perspectives of transcription factor EB (TFEB): mechanisms of regulation and association with disease. *Cell Death Differ.* **29**, 1433–1449 (2022). 35739255 10.1038/s41418-022-01028-6 PMC9345944

[39] Yoneshima, E. et al. The transcription factor EB (TFEB) regulates osteoblast differentiation through ATF4/CHOP-dependent pathway. *J. Cell Physiol.* **231**, 1321–1333 (2016). 26519689 10.1002/jcp.25235

[40] Ferron, M. et al. A RANKL-PKCbeta-TFEB signaling cascade is necessary for lysosomal biogenesis in osteoclasts. *Genes Dev.* **27**, 955–969 (2013). 23599343 10.1101/gad.213827.113 PMC3650231

[41] Gibault, F. et al. Non-photoinduced biological properties of verteporfin. *Curr. Med. Chem.* **23**, 1171–1184 (2016). 26980565 10.2174/0929867323666160316125048

[42] Wang, G., Ge, L., Liu, T., Zheng, Z. & Chen, L. The therapeutic potential of arctigenin against multiple human diseases: A mechanistic review. *Phytomedicine* **110**, 154647 (2023). 36628833 10.1016/j.phymed.2023.154647

[43] Shen, S. et al. Synthesis and biological evaluation of arctigenin ester and ether derivatives as activators of AMPK. *Bioorg. Med. Chem.* **21**, 3882–3893 (2013). 23673223 10.1016/j.bmc.2013.04.010

[44] Cheng, Y. H., Dong, J. C. & Bian, Q. Small molecules for mesenchymal stem cell fate determination. *World J. Stem Cells* **11**, 1084–1103 (2019). 31875870 10.4252/wjsc.v11.i12.1084 PMC6904864

[45] Weng, Z. et al. Mesenchymal stem/stromal cell senescence: hallmarks, mechanisms, and combating strategies. *Stem Cells Transl. Med.* **11**, 356–371 (2022). 35485439 10.1093/stcltm/szac004 PMC9052415

[46] Carrageta, D. F., Freire-Brito, L., Oliveira, P. F. & Alves, M. G. Evaluation of human spermatozoa mitochondrial membrane potential using the JC-1 Dye. *Curr. Protoc.* **2**, e531 (2022). 36066206 10.1002/cpz1.531

[47] Perelman, A. et al. JC-1: alternative excitation wavelengths facilitate mitochondrial membrane potential cytometry. *Cell Death Dis.* **3**, e430 (2012). 23171850 10.1038/cddis.2012.171 PMC3542606

[48] Ballabio, A. & Bonifacino, J. S. Lysosomes as dynamic regulators of cell and organismal homeostasis. *Nat. Rev. Mol. Cell Biol.* **21**, 101–118 (2019). 31768005 10.1038/s41580-019-0185-4

[49] Puertollano, R., Ferguson, S. M., Brugarolas, J. & Ballabio, A. The complex relationship between TFEB transcription factor phosphorylation and subcellular localization. *EMBO J.* **37**, e98804 (2018). 29764979 10.15252/embj.201798804 PMC5983138

[50] Qiu, D. et al. In vitro determination of osteo-adipogenic lineage choice of bone marrow stromal/stem cells (BMSCs). *Methods X* **12**, 102637 (2024). 10.1016/j.mex.2024.102637 PMC10912731 38445171

[51] Zhou, B. O., Yue, R., Murphy, M. M., Peyer, J. G. & Morrison, S. J. Leptin-receptor-expressing mesenchymal stromal cells represent the main source of bone formed by adult bone marrow. *Cell Stem Cell* **15**, 154–168 (2014). 24953181 10.1016/j.stem.2014.06.008 PMC4127103

[52] Shen, B. et al. A mechanosensitive peri-arteriolar niche for osteogenesis and lymphopoiesis. *Nature* **591**, 438–444 (2021). 33627868 10.1038/s41586-021-03298-5 PMC7979521

[53] Kaushik, S. et al. Autophagy and the hallmarks of aging. *Ageing Res. Rev.* **72**, 101468 (2021). 34563704 10.1016/j.arr.2021.101468 PMC8616816

[54] Choi, H. K. et al. Tsc1 Regulates the balance between osteoblast and adipocyte differentiation through autophagy/Notch1/β-Catenin cascade. *J. Bone Miner. Res.* **33**, 2021–2034 (2018). 29924882 10.1002/jbmr.3530 PMC6248888

[55] Ceccariglia, S., Cargnoni, A., Silini, A. R. & Parolini, O. Autophagy: a potential key contributor to the therapeutic action of mesenchymal stem cells. *Autophagy* **16**, 28–37 (2020). 31185790 10.1080/15548627.2019.1630223 PMC6984485

[56] Debnath, J., Gammoh, N. & Ryan, K. M. Autophagy and autophagy-related pathways in cancer. *Nat. Rev. Mol. Cell Biol.* **24**, 560–575 (2023). 36864290 10.1038/s41580-023-00585-z PMC9980873

[57] Luo, G. et al. Verteporfin attenuates trauma-induced heterotopic ossification of Achilles tendon by inhibiting osteogenesis and angiogenesis involving YAP/*β*-catenin signaling. *The FASEB Journal* **37**, e23057 (2023). 10.1096/fj.202300568R 37367700

[58] Malik, N. et al. Induction of lysosomal and mitochondrial biogenesis by AMPK phosphorylation of FNIP1. *Science* **380**, eabj5559 (2023). 37079666 10.1126/science.abj5559 PMC10794112

[59] Compston, J. E., McClung, M. R. & Leslie, W. D. Osteoporosis. *Lancet* **393**, 364–376 (2019). 30696576 10.1016/S0140-6736(18)32112-3

[60] Mo, C. et al. Single-cell transcriptomics of LepR-positive skeletal cells reveals heterogeneous stress-dependent stem and progenitor pools. *The EMBO journal* **41**, e108415 (2021). 10.15252/embj.2021108415 PMC8844986 34957577

[61] Tikhonova, A. N. et al. The bone marrow microenvironment at single-cell resolution. *Nature* **569**, 222–228 (2019). 30971824 10.1038/s41586-019-1104-8 PMC6607432

[62] Decker, M. et al. Leptin-receptor-expressing bone marrow stromal cells are myofibroblasts in primary myelofibrosis. *Nat. Cell Biol.* **19**, 677–688 (2017). 28481328 10.1038/ncb3530 PMC5801040

[63] Sarkaria, S. M. et al. Systematic dissection of coordinated stromal remodeling identifies Sox10^+^ glial cells as a therapeutic target in myelofibrosis. *Cell Stem Cell* **30**, 832–850.e836 (2023). 37267917 10.1016/j.stem.2023.05.002 PMC10240254

[64] Zhang, H. et al. The roles of bone remodeling in normal hematopoiesis and age-related hematological malignancies. *Bone Res.* **11**, 15 (2023). 36918531 10.1038/s41413-023-00249-w PMC10014945

[65] Lopez-Otin, C., Blasco, M. A., Partridge, L., Serrano, M. & Kroemer, G. The hallmarks of aging. *Cell* **153**, 1194–1217 (2013). 23746838 10.1016/j.cell.2013.05.039 PMC3836174

[66] Guo, J. et al. Aging and aging-related diseases: from molecular mechanisms to interventions and treatments. *Signal Transduct. Target Ther.* **7**, 391 (2022). 36522308 10.1038/s41392-022-01251-0 PMC9755275

[67] Li, X. et al. Inflammation and aging: signaling pathways and intervention therapies. *Signal Transduct. Target Ther.* **8**, 239 (2023). 37291105 10.1038/s41392-023-01502-8 PMC10248351

[68] Wang, T. & He, C. TNF-α and IL-6: the link between immune and bone system. *Curr. drug targets* **21**, 213–227 (2020). 31433756 10.2174/1389450120666190821161259

[69] Okamoto, K. & Takayanagi, H. Osteoimmunology. *Cold Spring Harb. Perspect. Med.* **9**, a031245 (2019). 10.1101/cshperspect.a031245 PMC6314075 29610150

[70] Tsukasaki, M. & Takayanagi, H. Osteoimmunology: evolving concepts in bone-immune interactions in health and disease. *Nat. Rev. Immunol.* **19**, 626–642 (2019). 31186549 10.1038/s41577-019-0178-8

[71] Luo, Z. et al. Mechano growth factor (MGF) and transforming growth factor (TGF)-β3 functionalized silk scaffolds enhance articular hyaline cartilage regeneration in rabbit model. *Biomaterials* **52**, 463–475 (2015). 25818452 10.1016/j.biomaterials.2015.01.001

[72] Wang, Z., Wei, D. & Xiao, H. in *Biological Aging: Methods and Protocols* (ed T. O. Tollefsbol) 135-144 (Humana Press, 2013).

[73] Li, H. et al. Mechano-growth factor enhances differentiation of bone marrow-derived mesenchymal stem cells. *Biotechnol. Lett.* **37**, 2341–2348 (2015). 26330369 10.1007/s10529-015-1915-0

[74] Shukla, S. K., Dasgupta, A., Mulder, S. E. & Singh, P. K. Molecular and physiological evaluation of pancreatic cancer-induced Cachexia. *Methods Mol. Biol.* **1882**, 321–333 (2019). 30378066 10.1007/978-1-4939-8879-2_28 PMC6709446

