# Supplementary material for: Rejuvenation of BMSCs senescence by pharmacological enhancement of TFEB-mediated autophagy alleviates aged-related bone loss and extends lifespan in middle aged mice
Source: Bone Res. 2024 Aug 21;12:45. doi: 10.1038/s41413-024-00351-7 (PMC11336217; doi:10.1038/s41413-024-00351-7)
Supplement: Supplementary file 1 — supplement data-clean version [file 41413_2024_351_MOESM1_ESM.docx]

Supplemental data for

**Rejuvenation of BMSCs senescence by pharmacological enhancement of TFEB-mediated autophagy alleviates aged-related bone loss and extends lifespan in middle aged mice**

Ziwei Luo ^a, *, #^, Wanyi Wei ^b, #^, Dawei Qiu ^c^, Zixia Su ^d^, Liangpu Liu ^a^,

Honghai Zhou ^a^, Hao Cui ^e, *^, Li Yang ^f^

^a^ College of Orthopedics, Guangxi University of Chinese Medicine, Nanning, 530200, Guangxi, P. R. China

^b^ Faculty of Chinese Medicine Science Guangxi University of Chinese Medicine, Nanning 530200, Guangxi, P. R. China

^c^ Department of Physical Education, Guangxi University of Chinese Medicine, Nanning 530200, Guangxi, P. R. China

^d^ Guangxi Key Laboratory of Efficacy Study on Chinese Materia Medica, Guangxi University of Chinese Medicine, Nanning, 530200, Guangxi, P. R. China

^e^ College of Pharmaceutical Sciences, Guangxi University of Chinese Medicine, Nanning 530200, Guangxi, P. R. China

^f^ Key Laboratory of Biorheological Science and Technology, Ministry of Education, Bioengineering College, Chongqing University, Chongqing 400030, P. R. China


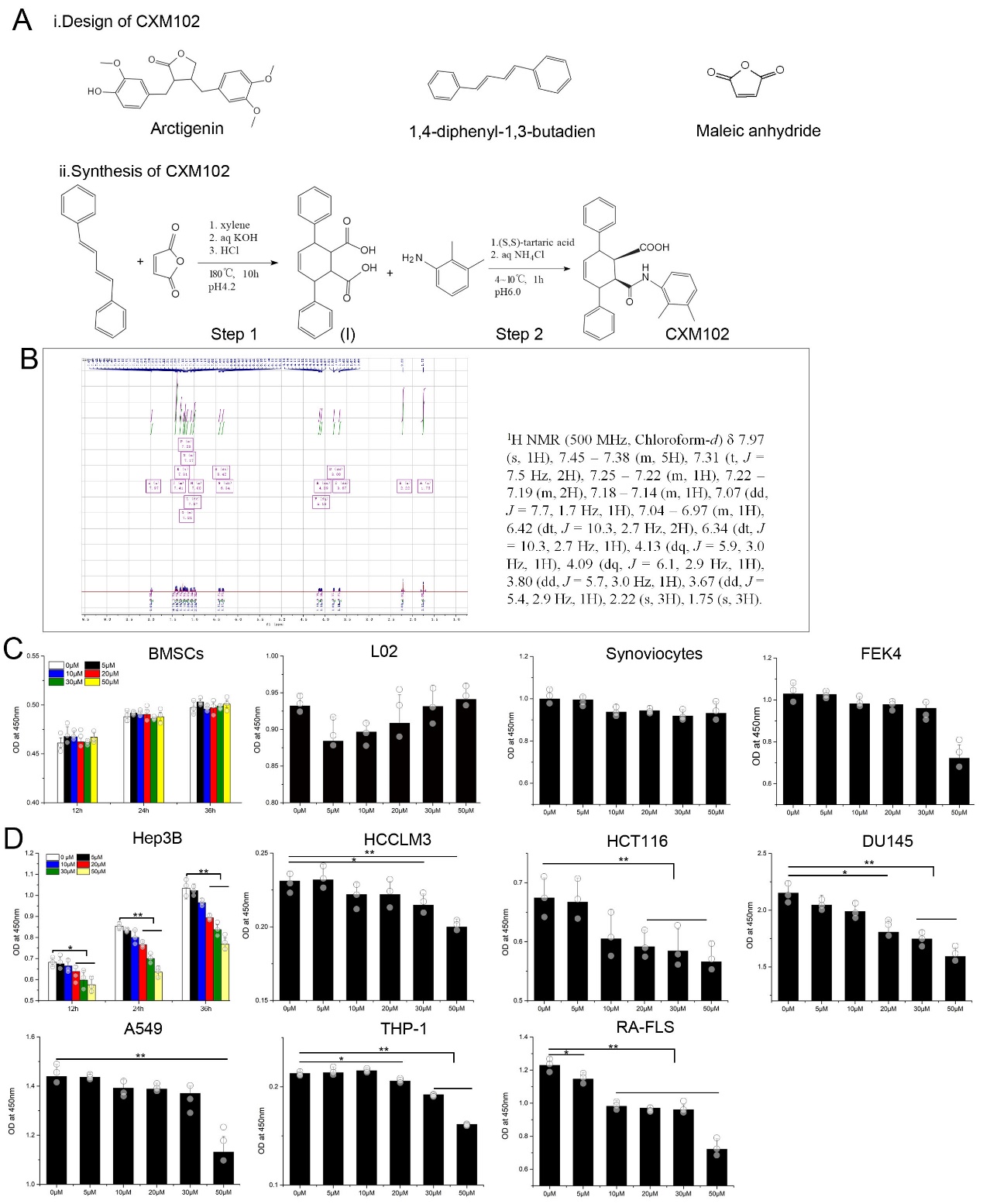


**Figure S1**. Synthesis of CXM102 and its cytotoxic effects on normal cells and cancer cells. **(A-B)** Brief description of the synthesis and hydrogen spectra of CXM102. (**C**) Effects of CXM102 on cell growth in human normal BMSCs, L02, synoviocytes and FEK4 cells. (**D)** Effects of CXM102 on cell growth in human cancer cell lines (Hep3B, HCCLM3, HCT116, DU145, A549 and THP-1) and rheumatoid arthritis fibroblast-like synoviocytes (RA-FLS). hBMSCs and Hep3B cells were determined at 12h, 24h and 36h after CXM102 treatment, while the other cells were determined at 12h. Results are presented as means ± SD, n ≥ 3. **p* < 0.05, ***p* < 0.01.


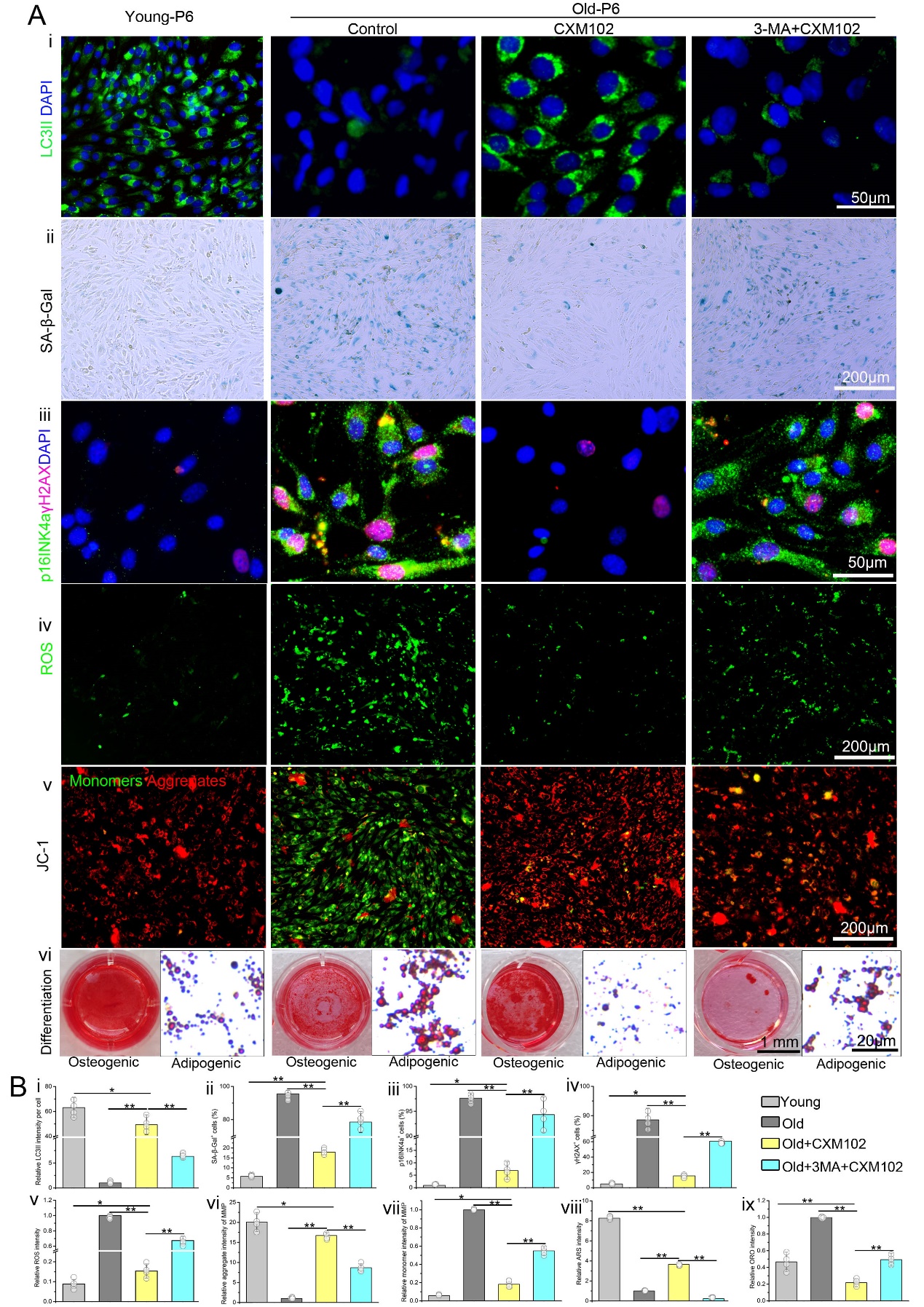


**Figure S2.** Pretreatment with 3-MA inhibits CXM102-mediated rejuvenation of aged hBMSCs. (**A**) Representative staining images of autophagosomes (LC3II), SA-β-gal, p16INK4a and γH2AX, ROS, JC-1 and osteo-adipogenic differentiation in young and old hBMSCs treated with CXM102 and/or 3-MA or not. (**B**) Quantitative analysis of LC3II puncta, SA-β-Gal, p16INK4a and γH2AX positive cells, ROS level, MMP and osteo-adipogenic differentiation in A. Results are presented as means ± SD, n =4. **p* < 0.05, ***p* < 0.01.


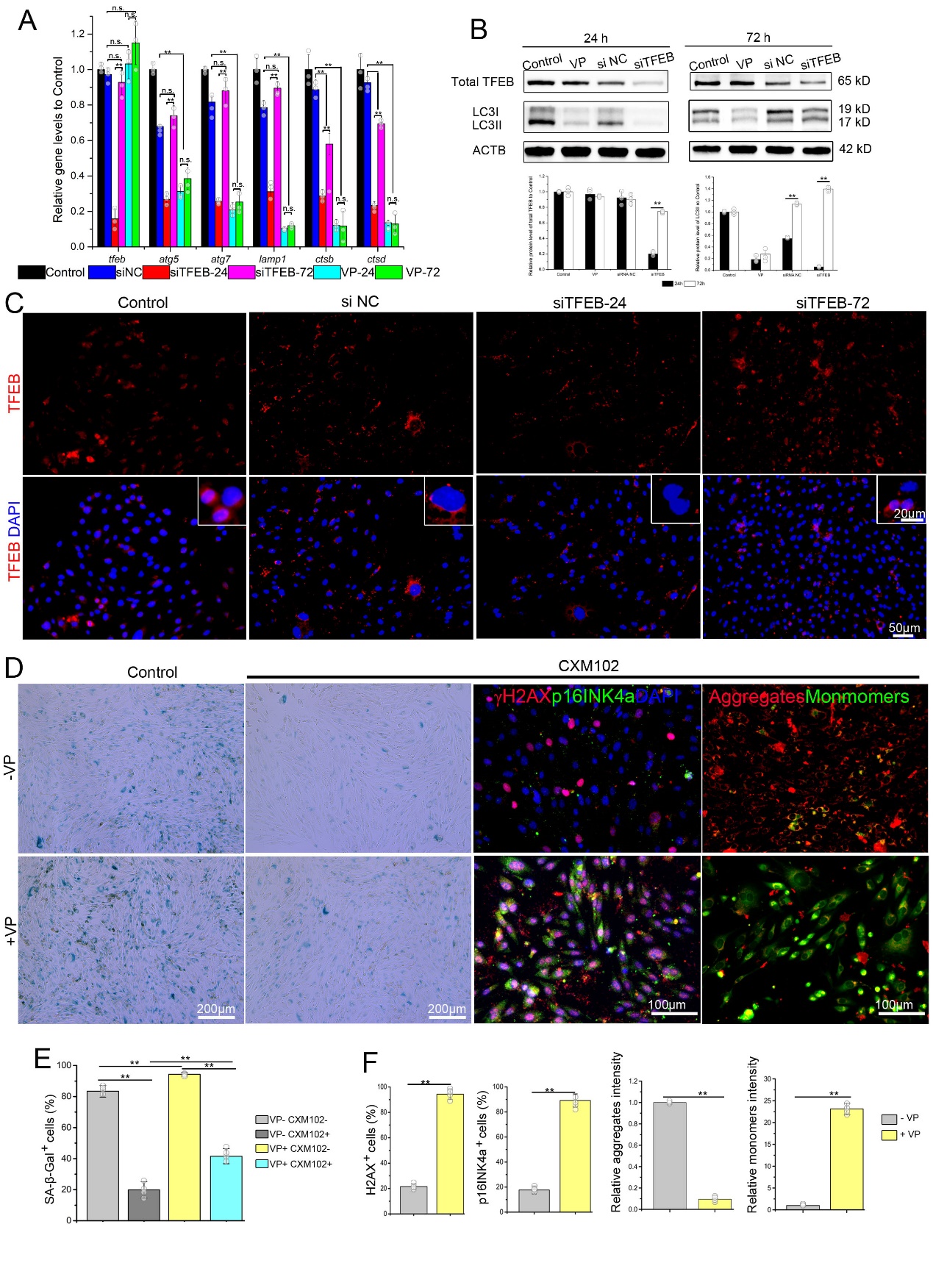


**Figure S3**. RNA interference downregulates total and nuclear expression of TFEB in short term, while verteporfin (VP) inhibits CXM102-induced rejuvenation of old hBMSCs. (**A**) Relative TFEB-targeting genes expression with short-term (24h) and long-term (72h) exposure to siRNA negative control (NC), siTFEB, and VP treatment. (**B**) Representative western blotting analysis of TFEB expression under the indicated condition. (**C**) Representative images of TFEB staining within 24 and 72 hours after siRNA transfection. (**D**) Representative images of SA-β-gal staining and double staining of p16INK4a and γH2AX in old hBMSCs treated with CXM102 and/or VP or not. (**E-F**) Quantitative analysis of SA-β-Gal (**E**), 16INK4a and γH2AX positive cells and relative MMP(**F**) in D. Results are presented as means ± SD, n ≥ 3. **p* < 0.05, ***p* < 0.01.


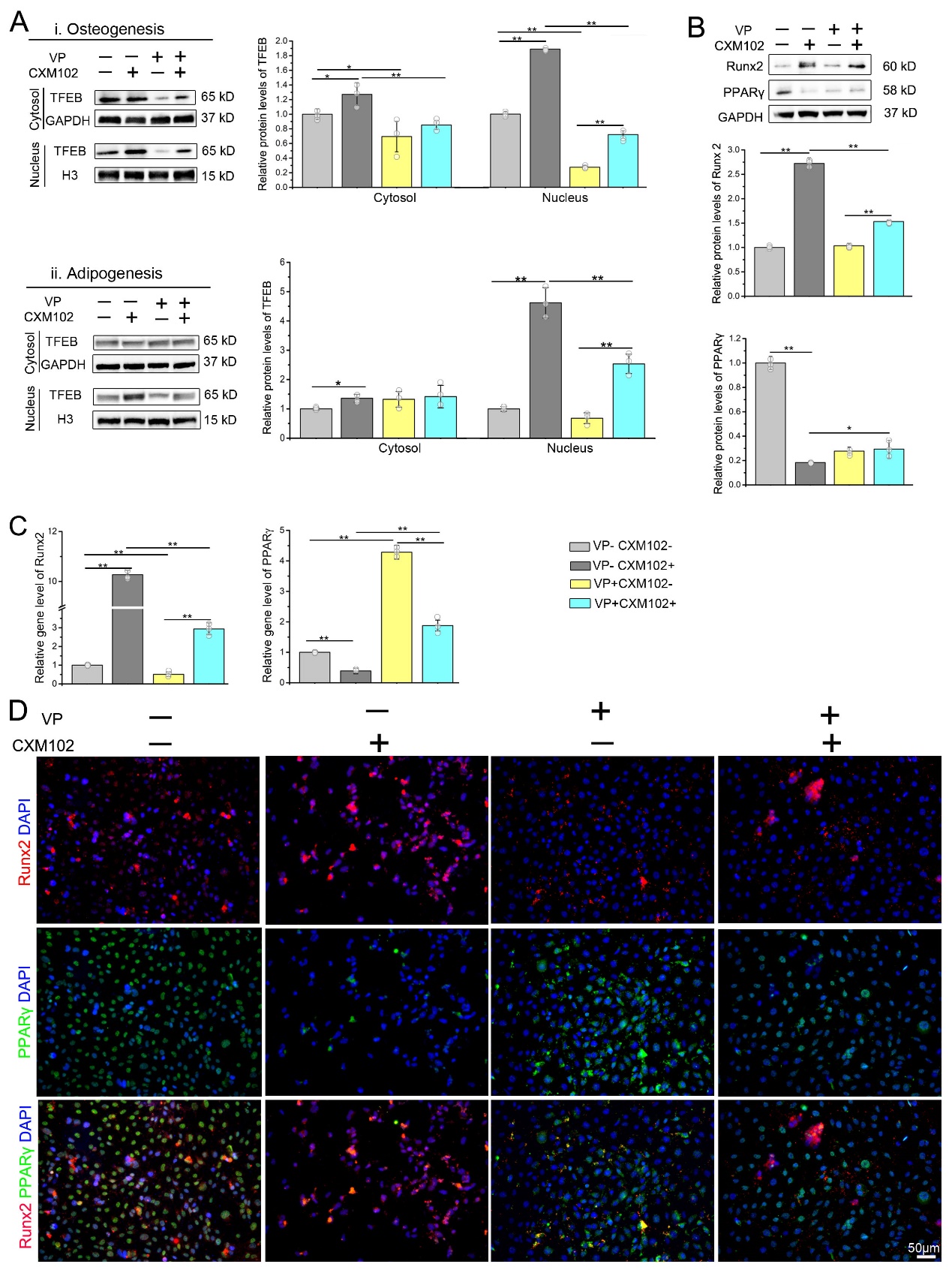


**Figure S4.** CXM102 promotes osteogenesis of hBMSCs through nuclear translocation of TFEB. (**A**) Cytosol and nuclear fraction of TFEB in young hBMSCs responded to osteogenic (i) and adipogenic (ii) induction as treated in Figure 4A. (**B**) Western blotting analysis of Runx2 and PPARγ as treated in Figure 4A. (**C-D**) Gene (**C**) and double-staining of Runx2 and PPARγ (**D**) analysis as treated in Figure 4B. Results are presented as means ± SD, n =3. **p* < 0.05, ***p* < 0.01.


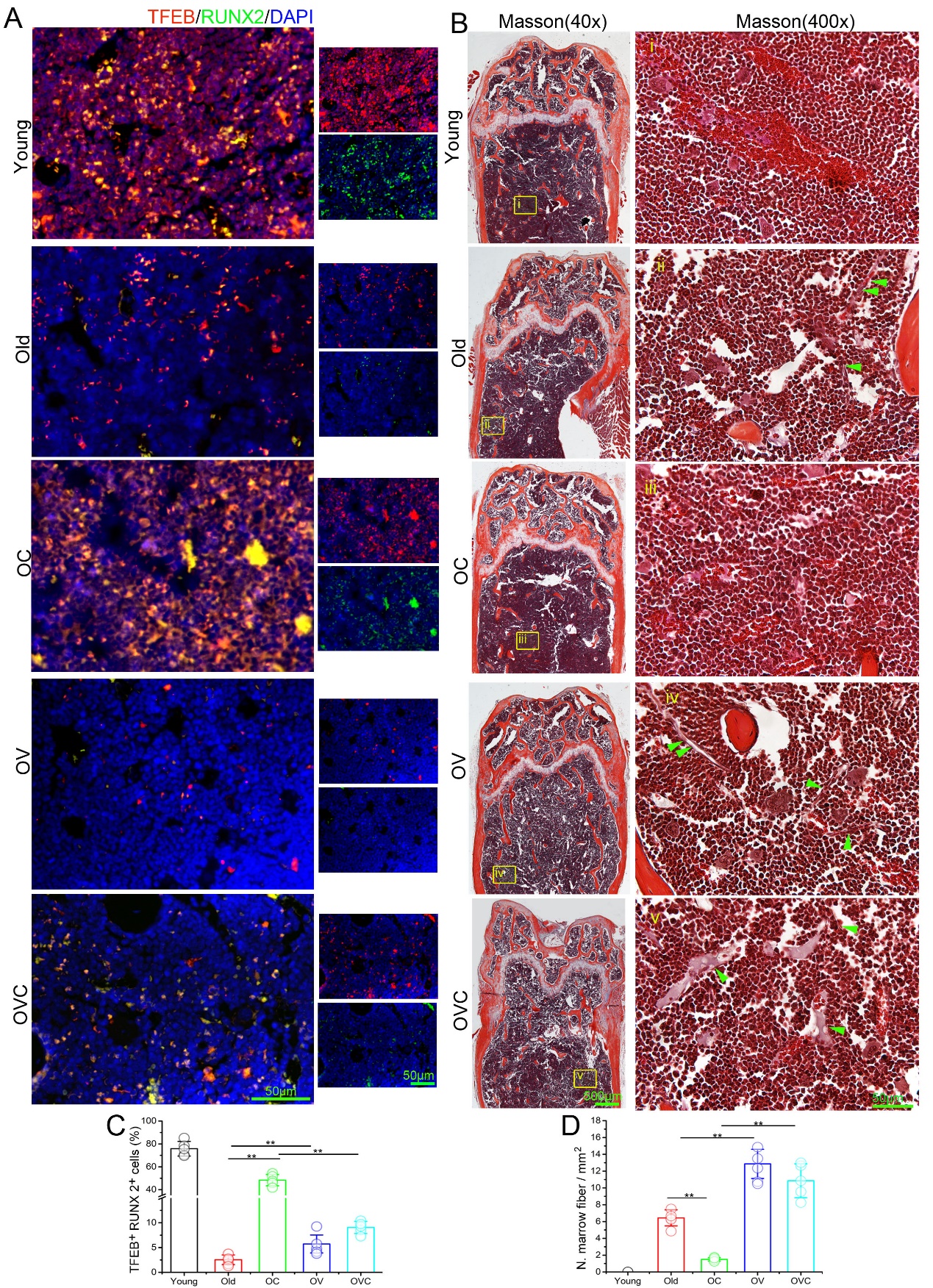


**Figure S5.** CXM102 increases osteoblasts formation and decreases myelofibrosis in old bone marrow. (**A-B**) Representative immunofluorescent images of double staining for TFEB and Runx2 (**A**) and Masson’s staining images (**B**) of bone marrow sections in each group of mice. (**C**) Quantitative analysis of TFEB and RUNX2 positive cells in A. (**D**) Quantitative analysis of myelofibrosis in bone marrow in B. Results are presented as means ± SD, n ≥ 3. **p* < 0.05, ***p* < 0.01.

**Table S1. Primary and secondary antibodies used in this study.**

| **Products** | **Company/**  **Brand** | **Catalog** | **Application**  **/Dilution** |
| --- | --- | --- | --- |
| **Primary antibodies** |  |  |  |
| TFEB | Abcam | ab2636 | IF/1:200  WB/1:1000 |
| LC3II | Abcam | ab192890 | IF/1:200  WB/1:1000 |
| LAMP1 | Invitrogen | 53-1071-82 | IF/1:200 |
| γH2AX(Ser139) | Invitrogen | MA1-2022 | IF/1:200 |
| P16INK4a | Affinity | AF5484 | IF/1:200 |
| Perilipin 1 | Abcam | ab3526 | IF/1:200 |
| Mouse anti-RUNX2 | Abnova | H00000860-M01 | IF/1:200 |
| Rabbit anti-RUNX2 | Abcam | ab192256 | IF/1:200 |
| PPARγ | Beyotime | AF7797 | IF/1:100 |
| LepR | Novus | AF497 | IF/1:200 |
| **Secondary antibodies** |  |  |  |
| AF488 Donkey anti-mouse IgG | Invitrogen | A21206 | IF/1:1000 |
| AF555 Donkey anti-mouse IgG | Invitrogen | A-31570 | IF/1:1000 |
| AF488 Goat anti-mouse IgG | Invitrogen | A11001 | IF/1:1000 |
| AF594 Donkey anti-rabbit IgG | Invitrogen | A-21207 | IF/1:1000 |
| AF555 Donkey anti-goat IgG | Invitrogen | A32816 | IF/1:1000 |
| 4',6-diamidino-2-phenylindole (DAPI) | Invitrogen | 62247 | 1 μg/ml |
| HRP-labeled Donkey Anti-Goat IgG | Beyotime | A0181 | IHC/1:100  WB/1:1000 |
| HRP-labeled Goat Anti-Rabbit IgG | Beyotime | A0208 | WB/1:1000 |
| HRP-labeled Goat Anti-Mouse IgG | Beyotime | A0216 | WB/1:1000 |
| Histone H3 | Beyotime | AH433 | WB/1:1000 |
| GAPDH | Beyotime | AG0122 | WB/1:1000 |
| β-acting (ACTB) | Beyotime | AF2815 | WB/1:1000 |

**Table S2. Primers used in this study.**

| Gene | Sequence |
| --- | --- |
| *Tfeb* | Forward: 5`-ATCACAAGCGAAGGTGACAT-3` |
|  | Reverse: 5`-GCAAGCATGCCTATTCGTGA-3` |
| *Atg5* | Forward: 5`-ATAGATGTGCTTCGAGATGTGT-3` |
|  | Reverse: 5`-CACTATGTCAGTTACGAACGTCA-3` |
| *Atg7* | Forward: 5`-ATGATCATCTGTAACTAGCCA-3` |
|  | Reverse: 5`-CACGGAAGCACCAACTTCAAC-3` |
| *Lamp1* | Forward: 5`-TCTCAGTGAACTACGACACCA -3` |
|  | Reverse: 5`-AGTGTATGTCCTCTTCCATAGC -3` |
| *Ctsb* | Forward: 5`-AGCCTTATGTACCGAGGACCT-3` |
|  | Reverse: 5`-GATGCAGATCAGTCAGAGA -3` |
| *Ctsd* | Forward: 5`-ATTCAGTACGTACATGATCAC-3` |
|  | Reverse: 5`-CGACACCTTGAGCGTGTAG -3` |
| *Gapdh* | Forward: 5`-АСАССGАСТССТССАССAТТ-3` |
|  | Reverse: 5`-TTACTCCTTGGAGCGCATGT -3` |
| *Runx2* | Forward-5′-TGACATCCCCATCCATCCAC-3′ |
|  | Reverse-5′-AGAAGTCAGAGGTGGCAGTG-3′ |
| *Pparγ* | Forward-5′-TATCACTGGAGATCTCCGCCAACAGC-3′ |
|  | Reverse-5′-GTCACGTTCTGACAGGACTGTGTGAC-3′ |
